# Evaluation of the potential therapeutic efficacy of *Cerastes cerastes* venom in acute experimental toxoplasmosis

**DOI:** 10.1186/s13071-025-07209-9

**Published:** 2026-01-24

**Authors:** Lobna A. El-Zawawy, Doaa E. Said, Rana Abdelghaffar, Nehal A. Khalil, Sara A. Abdel Salam

**Affiliations:** 1 Department of Medical Parasitology, Faculty of Medicine, Alexandria University, Alexandria, Egypt; 2 Department of Medical Biochemistry, Faculty of Medicine, Alexandria University, Alexandria, Egypt

**Keywords:** Antioxidant, *Cerastes cerastes* venom, Infectivity, Molecular, Ultrastructural, *Toxoplasma gondii*

## Abstract

**Background:**

The control of toxoplasmosis relies on conventional chemotherapeutics, which have hitherto unresolved concerns.

**Methods:**

Swiss albino mice were intraperitoneally (IP) infected with 5 × 10^3^ tachyzoites of RH HXGPRT( −) strain of *Toxoplasma gondii*, then IP treated with one-fourth lethal dose 50 (one-fourth LD50) of *Cerastes cerastes* venom (CCV) for three consecutive days (LD = 0.535 mg/kg). The anti-*Toxoplasma* activity of CCV was evaluated, for the first time, in immunocompetent (IC) and immunosuppressed (IS) mice via estimation of their mortality and survival time, microscopical counting of peritoneal tachyzoites, measurement of liver parasite burdens using quantitative real-time polymerase chain reaction (qRT–PCR), detection of infectivity, and ultrastructural changes of the treated tachyzoites. The safety of the used dose was biochemically assessed by measuring liver, kidney, and oxidative stress markers in serum.

**Results:**

CCV induced an insignificant reduction in mortality rate (MR) and a significant increase in survival time of mice. A statistically significant decrease in the mean peritoneal parasite burden with 89.8% and 90.8% reduction (%R) was observed in both IC and IS-treated subgroups compared with their controls, respectively. This reduction was consistent with 88% and 86% decrease in liver parasite load, respectively, and obvious ultrastructural alterations in treated tachyzoites. Concerning the infectivity study, the percent reduction was 78.8% and 85.5% in the peritoneal fluid and 71.1% and 60.4% in the liver tissues of IC and IS subgroups, respectively. The biochemical safety of the used dose and its high antioxidant activity were verified.

**Conclusions:**

Thus, one-fourth LD50 of CCV can be considered a promising, effective natural alternative to standard chemotherapy for acute toxoplasmosis.

**Graphical abstract:**

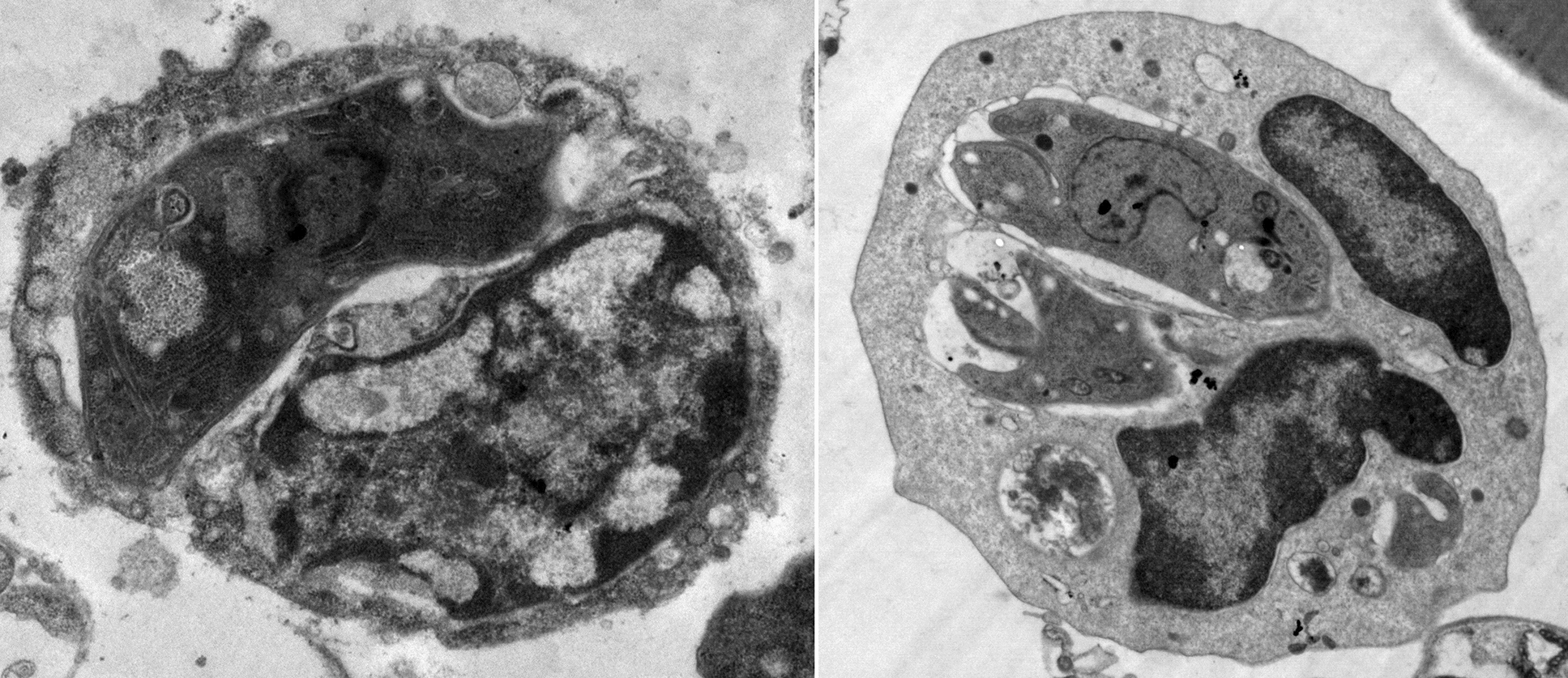

**Supplementary Information:**

The online version contains supplementary material available at 10.1186/s13071-025-07209-9.

## Background

Toxoplasmosis has been a major health problem impacting the human population worldwide. It is caused by the obligate intracellular protozoan *Toxoplasma gondii* (*T. gondii*). It can infect all warm-blooded animals, including humans, making it a remarkably ubiquitous parasitic organism worldwide [1]. Man is infected by accidental ingestion of sporulated oocysts in contaminated water and food, as well as through the consumption of raw or undercooked meat containing tissue cysts. [2]. Rapidly multiplying tachyzoites of *T. gondii* can also be transmitted by vertical transmission from mother to developing fetus, blood transfusion, solid organ transplantation, or unintentional exposure in a laboratory setting [3]. In most IC patients, the infection remains latent. However, immunodeficient patients and congenitally infected fetuses are at high risk for severe life-threatening consequences. As there is no successful vaccine against toxoplasmosis, effective chemotherapy constitutes the only alternative to control the disease in humans. [2]. A combination of sulfadiazine and pyrimethamine is the standard treatment for toxoplasmosis. Although this combination is highly effective against rapidly dividing tachyzoites, no significant activity was detected against the encysted bradyzoites. [4]. Besides, the failure of the combined drugs to cure chronic infection, they can cause severe side effects such as bone marrow suppression and hypersensitivity [5, 6]. Therefore, there is an imperative need for the discovery of effective natural therapeutic alternatives against toxoplasmosis in a quest to curtail the current chemotherapeutic concerns.

Zootherapy refers to the utilization of animal-derived therapeutic compounds in the treatment of human ailments [7]. Recently, a plethora of animal-derived natural products have gained much attention in the biomedical field [8]. Some of them proved their anti-*Toxoplasma* activity mainly against tachyzoites, such as bee products (honey and propolis), lactoferrin (derived from mammals’ milk), amphibian skin secretions of anuran frog, and fish oil omega-3 polyunsaturated fatty acids, as well as venoms of ants, bees, scorpions, and snakes [9–14].

Since ancient times, a plethora of snake venoms have been used for medicinal purposes [15]. The horned viper snake, *C. cerastes*, is one of the medically important species of venomous snakes that is widely distributed throughout Southwestern Asia to North Africa, inhabiting the sandy deserts [16]. Remarkably, *Cerastes cerastes* venom (CCV) serves as a set of diverse, effective, pharmacologically active molecules. The most abundant are zinc metalloproteases (MPs), followed by phospholipases A2 (PLA2s), L-amino acid oxidases (LAAOs), disintegrins, hyaluronidases, and serine proteases (SPs). Whereas, cysteine-rich secretory proteins (CRISPs) and C-type lectin-like molecules measured lower amounts of all venom toxins [17]. Owing to its salient attributes, it exhibits multiple biological activities against rheumatoid arthritis, assorted cancers, and bacteria [18–21]. Regarding the in vitro antiparasitic activity, CCV showed promising effects against *Schistosoma mansoni* (*S. mansoni*), *Trypanosoma cruzi*, and *Leishmania infantum* (*L. infantum*) [22, 23]. Furthermore, it has recently exhibited a potential in vivo activity against *S. mansoni* [16]. These promising antiparasitic effects inspired the authors to assess the anti-*Toxoplasma* activity of a safe dose of crude CCV against murine acute toxoplasmosis, which has not been previously reported. Therefore, this work was designed to evaluate the therapeutic effect of CCV in IC and IS mice infected with the RH HXGPRT(−) virulent strain of *T. gondii* via molecular, parasitological, ultrastructural, and biochemical studies.

## Methods

### Experimental animals

The 120 laboratory-bred Swiss strain albino male mice, weighing 20 ± 5 g, were purchased from the animal house of the Medical Parasitology Department, Faculty of Medicine, Alexandria University, Egypt. In compliance with the ARRIVE guidelines, they were maintained under the same standard caging conditions (24 °C ± 1 °C, 60–65% relative humidity and 12-h light and 12-h dark cycle). They were fed a regular diet with free access to water, and their bedding was changed daily.

### Parasite

The virulent RH HXGPRT(−) strain of *T. gondii* was maintained in the Medical Parasitology Department, Faculty of Medicine, Alexandria University, by serial IP passage of 10^4^ tachyzoites in Swiss strain albino mice every 5 days [24, 25].

### Drugs

Cyclophosphamide (Endoxan, Asta Medica AG, Germany) was purchased from a local pharmacy. While lyophilized crude CCV was procured from VACSERA, Giza, Egypt. One mg of lyophilized crude CCV was dissolved and diluted in distilled water. Both lyophilized and diluted venoms were kept at −20°C until used [17].

### Animal grouping and experimental design

The 96 mice out of 120 mice were equally divided into two main groups: IC (group I) and IS (group II). In group I, 48 IC mice were allocated into 36 control (CTL) (subgroup Ia) and 12 experimentally infected treated (INF–T) mice (subgroup Ib). Subgroup Ia was further subdivided into three subgroups, 12 mice each, as follows: subgroup Ia1, noninfected nontreated (NI–NT); subgroup Ia2, noninfected treated (NI–T); and subgroup Ia3, infected non-treated (INF–NT). In group II, 48 IS mice were allocated into 36 CTL (subgroup IIa) and 12 experimentally INF–T mice (subgroup IIb). Subgroup IIa was further subdivided into three subgroups, 12 mice each, as follows: subgroup IIa1, NI–NT; subgroup IIa2, NI–T; and subgroup IIa3, INF–NT. The remaining 24 mice were used to estimate the infectivity of the animals. To induce immunosuppression in IS subgroups, each mouse received two IP doses of cyclophosphamide (70 mg/kg each) separated by a week [26].

Each mouse in INF subgroups Ia3, Ib, IIa3, and IIb was IP infected with 5 × 10^3^ tachyzoites of the RH HXGPRT(−) virulent strain of *T. gondii*. The IS mice in INF subgroups IIa3 and IIb were infected 48 h after administration of the second dose of cyclophosphamide [25].

The LD of CCV was determined to be 0.535 mg venom per kilogram body weight [21]. A pilot study was performed to adjust the schedule of venom administration. The minimal effective safe dose (one-fourth LD50) and duration (three consecutive days) that had efficiently reduced the parasite burden were chosen (Additional file 1). It was IP administered to the INF–T mice (subgroups Ib and IIb) for three consecutive days, starting 6 h after the infection [27]. A similar schedule of venom administration was implemented for the NI–T mice (subgroups Ia2 and IIa2).

Blood was drawn on the day of sacrifice, seventh day post infection (dpi), from the jugular veins of six out of 12 mice from each INF subgroup after being anesthetized with 40 mg/kg of thiopental sodium and sacrificed by cervical dislocation thereafter [28]. Similarly, blood was collected from half of the mice in the NI–T subgroups on the seventh day after the first dose of CCV, and then they were sacrificed. Peritoneal fluid and liver specimens were collected separately from each mouse. The remaining mice from all subgroups were observed daily to estimate their survival time.

On the sacrifice day, *T. gondii* tachyzoites collected from the peritoneal fluid of each INF subgroup (Ia3, Ib, IIa3, and IIb) were reinjected IP into another 24 naive mice (subgroups Ia3x, Ibx, IIa3x, and IIbx, respectively, six mice for each subgroup) to be used in the infectivity study [24].

### Assessment of anti-*Toxoplasma* therapeutic efficacy of venom

### Molecular study

#### Preparation of tissue and positive control samples

Liver specimens were collected on the day of sacrifice from each mouse in the INF subgroups (Ia3, Ib, IIa3, and IIb) and the infectivity study subgroups (Ia3x, Ibx, IIax3, and IIbx), as well as the NI mice (as a negative control) under aseptic conditions. Only 25 mg of each aseptically collected specimen was washed, wiped, and stored at −80 °C until used for DNA extraction.

To prepare a positive control sample, *T. gondii* tachyzoites from peritoneal exudates of INF–NT mice (subgroup Ia3) were collected and counted, and a specimen of 1 × 10⁷ tachyzoites was prepared and stored at −80 °C until used for DNA extraction.

#### DNA extraction

**Fig. 1:**
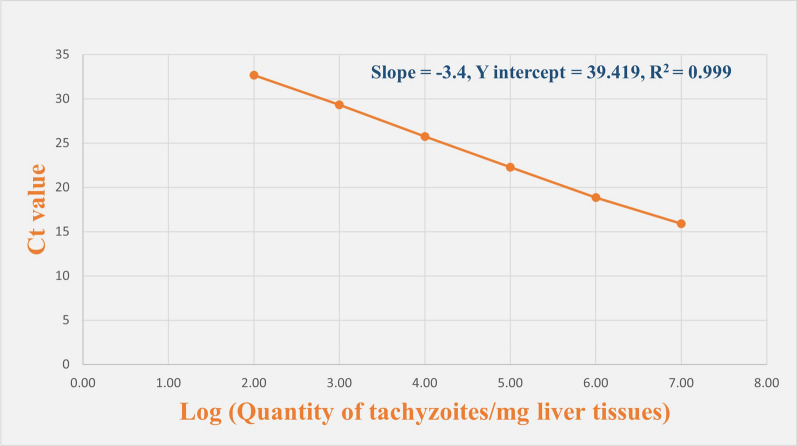
The standard curve for quantification of *T. gondii* tachyzoites. *Ct* cycle threshold, *R*^*2*^ correlation coefficient

Total genomic DNA was extracted from both liver specimens and *T. gondii* tachyzoites using DNeasy Blood and Tissue DNA kit (cat. no. 69504, QIAGEN™, Hilden, Germany), a rapid and high-quality silica-based, phenol- and chloroform-free spin-column extraction of DNA, according to the manufacturer’s instructions. The suspension containing tachyzoites’ DNA was quantified, assessed using a NanoDrop 2000 spectrophotometer (Thermo Scientific, USA), a microvolume ultraviolet (UV)–visible light spectrophotometer featuring a patented sample retention system with full spectral output (190–840 nm), and then serially diluted (ranging from 1 × 10^7^ to 1 × 10^2^ tachyzoite DNA). DNA serial dilutions were used to establish a standard curve using Applied Biosystem StepOne™ qRT-PCR software, a fast (< 40 min) software with standard runs (< 2 h) configured as shown in Fig. 1. The average cycle threshold (Ct) values were plotted on the *y* axis versus the quantity of tachyzoite logarithms (logs) in each dilution on the *x* axis. The efficiency of the qRT–PCR reaction was determined using the slope of the regression line in the standard curve according to the equation [29]

$${\text{Efficiency }}\% \, = \, \left( {{1}0^{{ - {1}/{\mathrm{slope}}}} - {1}} \right){\text{ x 1}}00$$

#### DNA amplification and quantification

The absolute fluorescence quantitative detection of DNA of *T. gondii* tachyzoites in liver specimens of infected mice was carried out with Step One™ qRT–PCR assay using Maxima SYBR Green master mix (cat. no. K0231, Thermo Fisher Scientific, USA), a ready-to-use 2× solution containing Maxima Hot Start Taq DNA Polymerase and ROX passive reference dye for high-specificity, and *T. gondii* B1 gene forward (5′-TCCCCTCTGCTGGCGAAAAGT-3′) and reverse primers (5′-AGCGTTCGTGGTCAACTATCGATT G-3′) (GenBank, AF179871.1, Invitrogen) [30, 31]. Each qRT–PCR run included positive and negative controls. A three-step cycling protocol was performed using the Step One™ qRT–PCR system (Applied Biosystems, USA).

The Ct value of each tested sample was obtained by Step One™ qRT–PCR software and used to calculate the quantities of tachyzoites according to the established standard curve. The results were expressed as *T. gondii* tachyzoites per milligram of liver tissue.

### Parasitological study

#### Mice observation

Mice were observed daily to detect any changes in behavior and food consumption till their spontaneous death.

#### Estimation of mortality rate

The dead mice in each experimental subgroup were counted on the day of sacrifice in comparison with their corresponding CTLs, and MR was assessed for each subgroup.

### Estimation of survival time

The remaining mice of each subgroup (6 out of 12 mice) that survived after the sacrifice day were observed daily to determine the survival percentage over time using the Kaplan–Meier curve [32].

#### Measurement of peritoneal parasite burden

The peritoneal fluid containing tachyzoites was collected from each mouse in the INF subgroups on the day of sacrifice. The mean number of extracellular tachyzoites was calculated per milliliter of peritoneal fluid in each experimental INF–T subgroup (Ib and IIb) and compared with their corresponding CTL subgroups (Ia3 and IIa3, respectively)[33]. Then, the percent reduction in the mean peritoneal parasite burden was estimated.

#### Estimation of animal infectivity

On the seventh dpi, mice of the infectivity study were sacrificed, and the effect of the CCV on the mice infectivity was assessed by estimating the infectivity rate (IR) and calculating the mean number of extracellular tachyzoites per ml of peritoneal fluid. The parasite load in liver tissues was calculated using qRT–PCR as mentioned before.

### Ultrastructural study

Tachyzoites obtained from peritoneal exudates of mice in the INF subgroups were centrifuged. For scanning electron microscopy (SEM), the resulting pellets were fixed in cold 2% glutaraldehyde in phosphate buffer (pH 7.4) overnight at 4 ℃. Then, tachyzoites were postfixed in 2% osmium tetroxide (OsO_4_) in 4-(2-hydroxyethyl)-1-piperazineethanesulfonic acid (HEPES) buffer for 2 h at room temperature, washed thoroughly with the same buffer, dehydrated in an ascending ethanol series, dried with *t*-butyl alcohol, and gold-coated in a sputter coater under vacuum to study the topographic changes using SEM (JEOL. JSM-IT200, Japan). It is a high-performance instrument featuring a high-resolution tungsten filament electron gun capable of high-resolution imaging below 10 nm in both low and high vacuum modes, and equipped with secondary and backscattered electron detectors [34].

For transmission electron microscopy (TEM), the tachyzoite pellets were fixed in cold 2.5% glutaraldehyde in a sodium cacodylate buffer (pH 7.4) for 3 h at 4 ℃. After that, tachyzoites were postfixed in 1% OsO_4_ in the HEPES buffer for 90 min at 4 ℃, washed thoroughly with the buffer, dehydrated in an ascending series of ethanol, and embedded in Epon 812 resin (Nisshin EM, Tokyo, Japan). The 80-nm-thick sections were doubly stained with uranyl acetate and lead citrate before examination through TEM (JEOL JEM-1400 plus, Japan). It is a 120 kV TEM designed for high-contrast and high-resolution imaging (down to 0.38 nm point resolution), and capabilities for seamless imaging from low to high magnifications (×10 to 1.2 M) [34].

### Biochemical study

The blood samples from each mouse in all studied subgroups were centrifuged, and serum samples were separated and kept at −20 °C. To verify the safety of the used dose of CCV, liver function markers [alanine transaminase (ALT) and aspartate transaminase (AST)] and kidney function markers (urea and creatinine) were measured using Jaffe and double enzymatic reactions, respectively. Whilst oxidative stress markers, malondialdehyde (MDA) and reduced glutathione (GSH), were colorimetrically assayed using commercial kits (cat. nos. 2529 and 2511, respectively, Biodiagnostic, Egypt) at 532 and 405 nm, respectively. The absorbance readings were obtained using the semi-automated photometer, Humalyzer Junior Spectrophotometer (Human Diagnostics, Germany).

## Statistical analysis

All used datasets were created in spreadsheets by Microsoft Excel software and uploaded to RStudio (R version 4.4.2, 2024) as CSV files for both descriptive and statistical analysis by the R programming language. A descriptive analysis for all variables was performed using graphs. Fisher exact test was applied for categorical data; more than 20% of cells had an expected count of less than five. The Shapiro–Wilk test was used to test the normality of numerical variables. For normally distributed quantitative variables, the Student *t*-test (two-sample *t*-test) was used to compare two studied groups. For not normally distributed data, the Kruskal–Wallis test was used to compare three or more subgroups, and the Wilcoxon rank sum test was applied for adjusted pairwise comparison between subgroups. Data visualization was presented in bar plots for categorical data and box plots for numerical continuous data. All study subgroups were independent. The Kaplan–Meier survival curve was computed using Graphpad Prism 8 (GraphPad Software Inc., USA), and the log-rank (Mantel–Cox) test was utilized for comparing the survival times of the studied subgroups. Statistical significance of the results obtained was judged at the 5% level.

The MR, percent reduction in the mean parasite burden, and IR were estimated by the following equations: [24, 35]

$$\text{MR }=\frac{\text{The number of dead mice at the sacrifice date}}{\text{The number of mice at the beginning of the experiment }} \times 100$$

$${\%R }=\frac{\mathrm{C}-\mathrm{T}}{\mathrm{C}} \times 100$$

*C*: mean number of tachyzoites in each INF–NT CTL subgroup.

*T*: mean number of tachyzoites in each INF–T experimental subgroup

$$\mathrm{IR}=\frac{\text{The number of infected mice on the sacrifice day}}{\text{The number of mice at the time of infection }} \times 100$$

## Results

### Molecular study

#### Quantification of *T. gondii* tachyzoites in liver

The qRT–PCR assay showed a high R^2^ value (0.999) and a slope of −3.4, corresponding to a qRT–PCR efficiency of 96.8%.

**Fig. 2:**
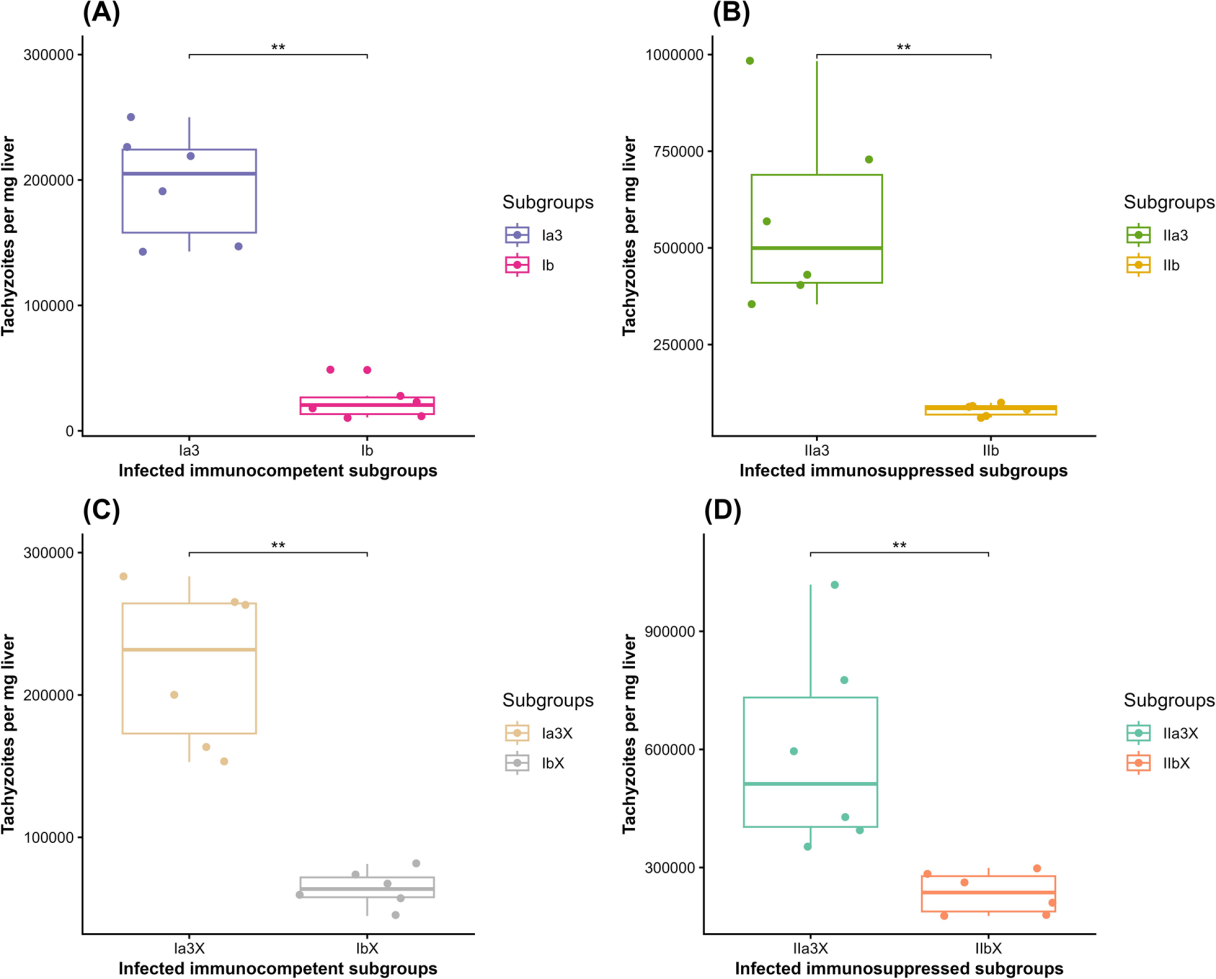
Boxplots demonstrating the effect of CCV on tachyzoite quantity per milligram liver tissues of INF–T IC and IS mice in comparison with their corresponding INF–NT CTLs using qRT–PCR: (**A**) IC mice; (**B**) IS mice; (**C**) IC mice of the infectivity study; (**D**) IS mice of the infectivity study. Ia3, INF–NT IC subgroup; Ib, INF–T IC subgroup; IIa3, INF–NT IS subgroup; IIb, INF–T IS subgroup; Ia3x, INF with tachyzoites from subgroup Ia3; Ibx, INF with tachyzoites from subgroup Ib; IIa3x, INF with tachyzoites from subgroup IIa3; and IIbx, INF with tachyzoites from subgroup IIb. **Statistically significant at *P* < 0.001 alpha level

In IC mice, the mean tachyzoite quantity was statistically significantly lower in the experimental INF–T subgroup Ib (23.317 × 10^3^ ± 13.969) compared with the INF–NT CTL subgroup Ia3 (196.000 × 10^3^ ± 43.7721) with 88%R (Wilcoxon rank sum test, *Z* = 36, *P* = 0.0022) (Fig. 2A). Similarly, in IS mice, the mean tachyzoite quantity was statistically significantly lower in the experimental INF–T subgroup IIb (81.283 × 10^3^ ± 15.276) compared with the INF–NT CTL subgroup IIa3 (578.000 × 10^3^ ± 240.671) with 86%R (Wilcoxon rank sum test, *Z* = 36, *P* = 0.0022) (Fig. 2B).

Regarding the infectivity study, there was a statistically significant reduction (71.1%) in the mean tachyzoite quantity in the experimental infectivity IC subgroup Ibx (64.017 × 10^3^ ± 12.883) compared with the corresponding CTL subgroup Ia3x (221.383 × 10^3^ ± 56.359) (Wilcoxon rank sum test, *Z* = 36, *P* = 0.0022) (Fig. 2C). Similarly, there was a statistically significant reduction (60.4%) in the mean tachyzoite quantity in the experimental infectivity IS subgroup IIbx (235.500 × 10^3^ ± 53.08) compared with the corresponding CTL subgroup IIa3x (594.869 × 10^3^ ± 26.021) (Wilcoxon rank sum test, *Z* = 36, *P* = 0.0022) (Fig. 2D).

### Parasitological study

#### Mice observation

Concerning the IC group, both NI–T and INF–T mice (subgroups Ia2 and Ib, respectively) were healthy with normal food intake and behavior when compared with those of NI–NT (subgroup Ia1). A decrease in food intake and lethargic behavior were observed in the INF–NT mice (subgroup Ia3). On the other hand, both NI–NT and NI–T IS mice (subgroups IIa1 and IIa2, respectively) showed a mild decrease in food intake and slight hair loss. Whereas the INF–NT IS mice (subgroup IIa3) showed a decrease in food intake, lethargic behavior with ruffled fur, hair loss, and a hunched back. Less pronounced alterations were noticed in the INF–T mice (subgroup IIb).

#### Estimation of MR

**Fig. 3:**
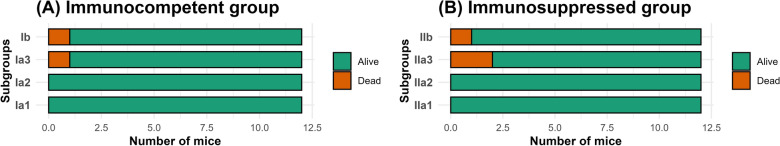
Bar plot showing the effect of CCV on the MR of the INF–T IC and IS mice in comparison with their corresponding CTLs: Ia1, NI–NT IC subgroup; Ia2, NI–T IC subgroup; Ia3, INF–NT IC subgroup; Ib, INF–T IC subgroup; IIa1, NI–NT IS subgroup; IIa2, NI–T IS subgroup; IIa3, INF–NT IS subgroup; and IIb, INF–T IS subgroup

In the IC mice, the MR was 8.3% in both INF–NT and INF–T (subgroups Ia3 and Ib, respectively). On the other hand, the MR in the IS mice was 16.7% and 8.3% in the INF–NT (subgroup IIa3) and the INF–T (subgroup IIb), respectively. In both IC and IS groups, there was a statistically insignificant difference in the MR between the experimental subgroups and their corresponding CTLs, as well as between the CTL subgroups (Fisher’s exact test, *P* = 1). Noticeably, no deaths were observed among the NI–T mice, whether IC or IS (Fig. 3).

#### Estimation of survival time

**Fig. 4:**
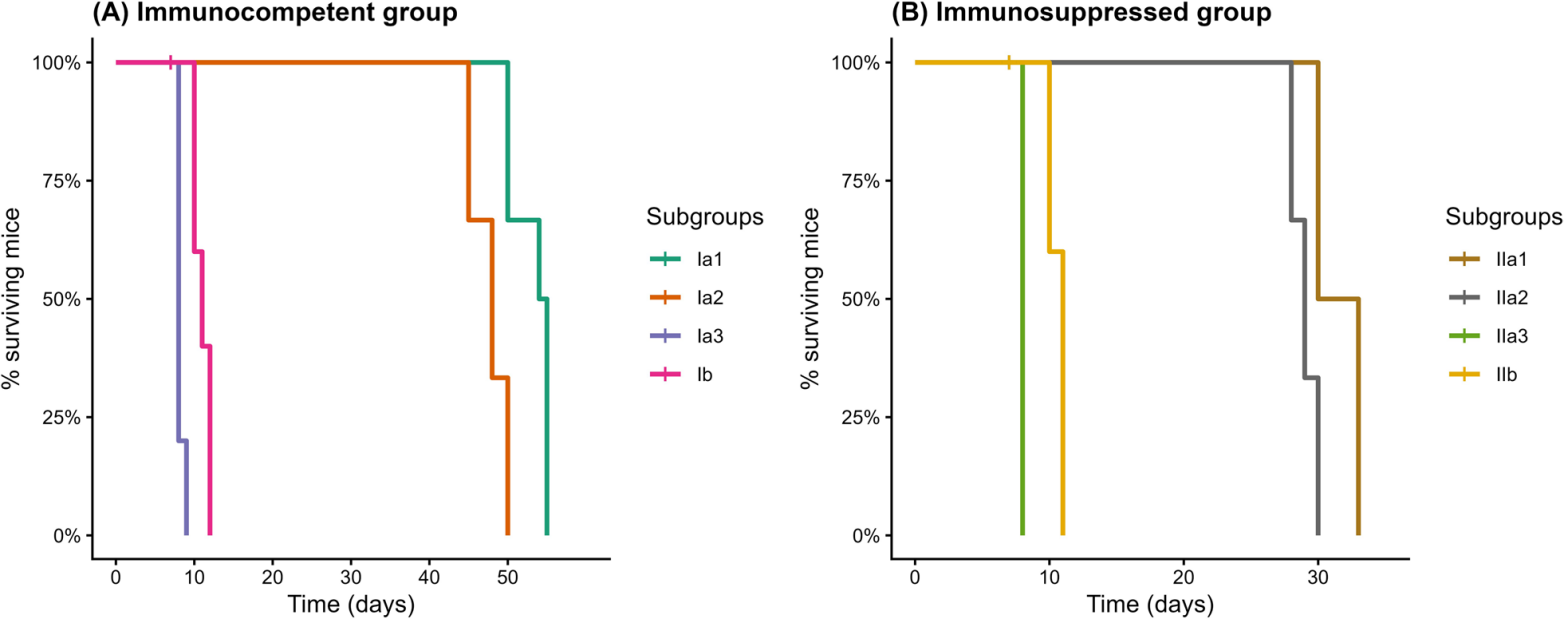
Kaplan–Meier survival curve showing the effect of CCV on the survival time of the INF–T IC and IS mice in comparison to their corresponding CTLs: Ia1, NI–NT IC subgroup; Ia2, NI–T IC subgroup; Ia3, INF–NT IC subgroup; Ib, INF–T IC subgroup; IIa1, NI–NT IS subgroup; IIa2, NI–T IS subgroup; IIa3, INF–NT IS subgroup; and IIb, INF–T IS subgroup

As depicted in Fig. 4, there was a statistically significant decrease in the mean survival time in the experimental subgroup Ib (11 ± 1 days) compared to their corresponding CTL subgroups Ia1 (53.17 ± 2.5 days) and Ia2 (47.67 ± 2.3 days) (log rank test, [*X*^*2*^ = 7.41, d.f. = 1, *P* = 0.0064], each), as well as between subgroup IIb (10.6 ± 0.5 days) and their corresponding CTL subgroups IIa1 (31.5 ± 1.6 days) and IIa2 (29.00 ± 0.9 days) (log rank test, [*X*^*2*^ = 6.82, d.f. = 1, *P* = 0.0089], each). Whereas there was a statistically significant increase in the mean survival time in the experimental subgroups Ib and IIb compared with the corresponding CTL subgroups Ia3 (8.2 ± 0.4 days) and IIa3 (8.00 ± 0.0 days), respectively (log rank test, [*X*^*2*^ = 0.20, d.f. = 1, *P* = 0.0096], [*X*^*2*^ = 0.18, d.f. = 1, *P* = 0.0281]).

#### Measurement of peritoneal parasite burden

**Fig. 5:**
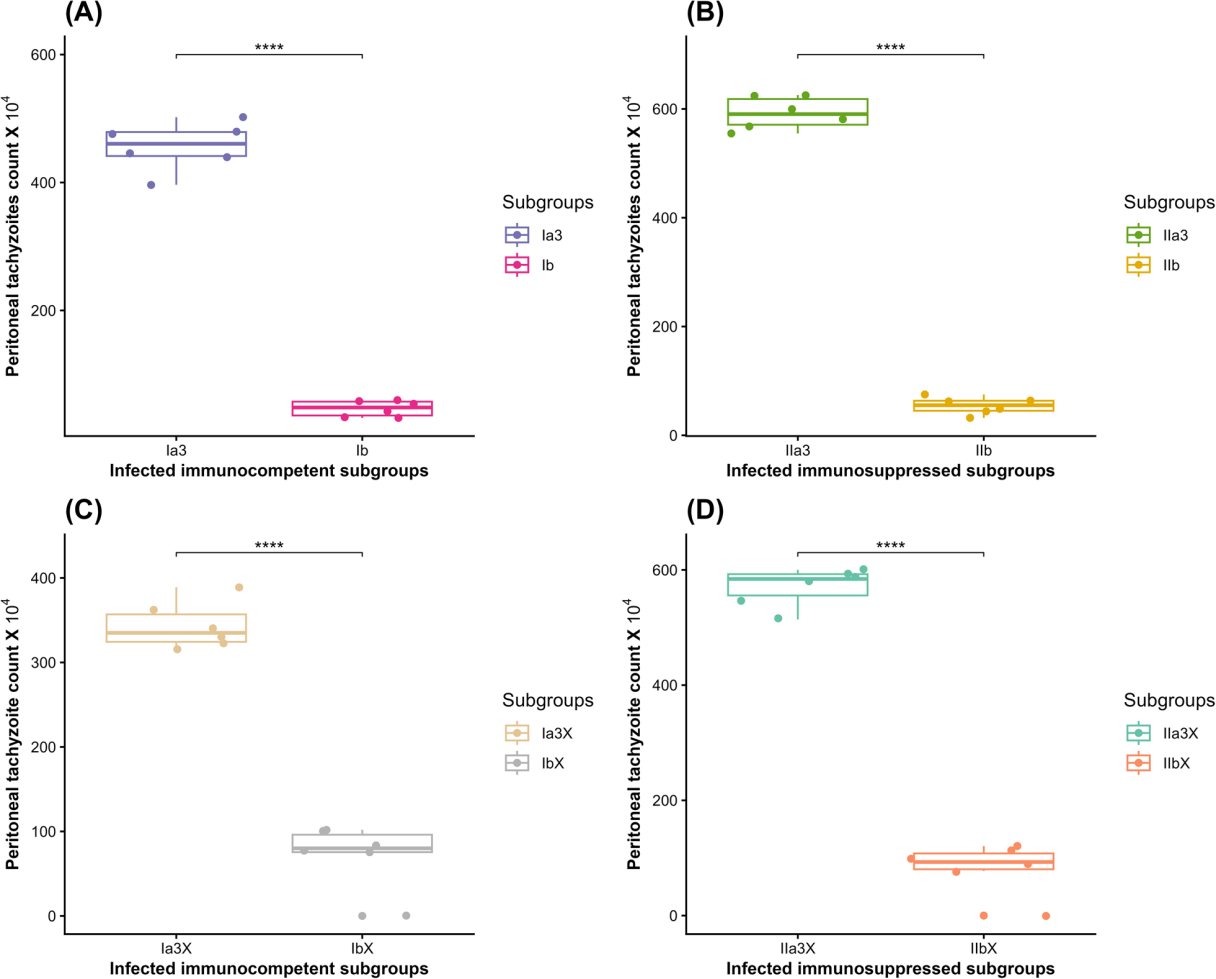
Boxplots demonstrating the effect of CCV on the peritoneal parasite burden in the INF–T IC and IS mice in comparison to their corresponding INF–NT CTL: (**A**) IC mice; (**B**) IS mice; (**C**) IC mice of the infectivity study; (**D**) IS mice of the infectivity study; Ia3, INF–NT IC subgroup; Ib, INF–T IC subgroup; IIa3, INF–NT IS subgroup; IIb, INF–T IS subgroup; Ia3x, INF with tachyzoites from subgroup Ia3; Ibx, INF with tachyzoites from subgroup Ib; IIa3x, INF with tachyzoites from subgroup IIa3; and IIbx, INF with tachyzoites from subgroup IIb. ****Statistically significant at *P* < 0.0001 alpha level

In IC mice, there was a statistically significant reduction (89.8%) in the mean parasite burden upon comparing experimental subgroup Ib (46.75 × 10^4^ ± 12.5) to the CTL subgroup Ia3 (456.6 × 10^4^ ± 37.4) (*t*-test, *t* = 25.5, d.f. = 6.1, *P* < 0.001) (Fig. 5A). Similarly, in the IS subgroups, there was a statistically significant reduction (90.8%) in the mean parasite burden when comparing experimental subgroup IIb (54.25 × 10^4^ ± 15.6) with the CTL subgroup IIa3 (592.2 × 10^4^ ± 29.4) (*t*-test, *t* = 39.6, d.f. = 7.62, *P* < 0.001) (Fig. 5B).

Regarding the infectivity study, there was a statistically significant reduction (78.8%) in the mean peritoneal parasite burden upon comparing the INF–T IC subgroup Ibx (72.92 × 10^4^ ± 37.5) to the INF–NT subgroup Ia3x (343.2 × 10^4^ ± 27.9) (*t*-test, *t* = 14.2, d.f. = 9.22, *P* < 0.001) (Fig. 5C). Whereas there was a statistically significant reduction (85.8%) in the mean parasite burden in the INF–T IS subgroup IIbx (82.58 × 10^4^ ± 43.3) compared with the INF–NT subgroup IIa3x (570.7 × 10^4^ ± 33.3) (*t*-test, *t* = 21.9, d.f. = 9.39, *P* < 0.001) (Fig. 5D).

#### Estimation of animal infectivity

No statistically significant difference was observed in the IR between the INF–NT (100%) and the INF–T (83.3%) subgroups, whether IC or IS (Fisher’s exact test, *P* = 1, each). The mean parasite burden in both peritoneal fluid and liver tissues for the infectivity study subgroups has been previously stated.

### Ultrastructural study

#### SEM

**Fig. 6:**
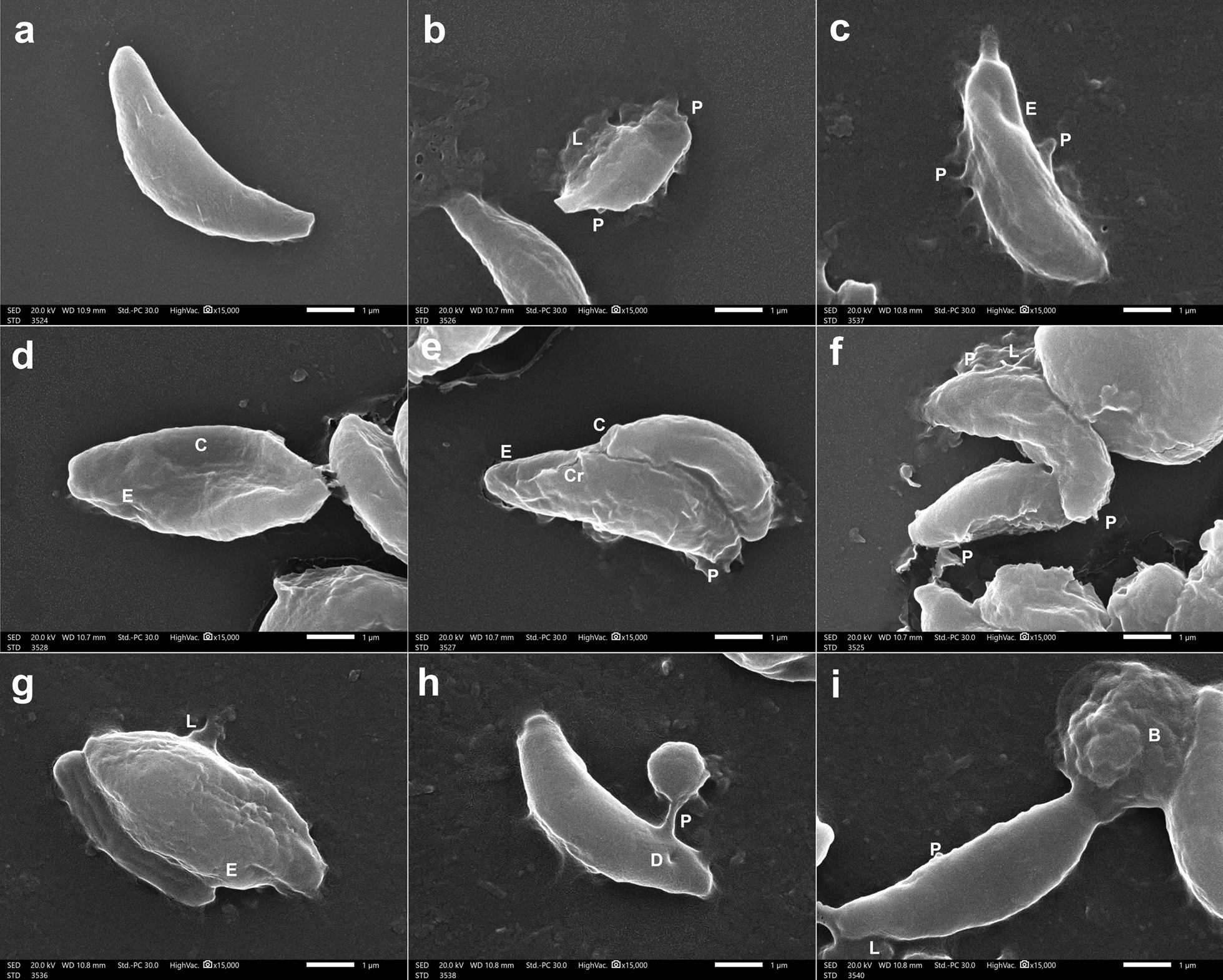
SEM of *T. gondii* tachyzoites retrieved from the peritoneal fluid of INF IC mice (×15,000): (**a**) NT tachyzoite (subgroup Ia3) showing an average-size and crescent-shaped with a completely smooth, regular surface. (b-i) CCV-T tachyzoites (subgroup Ib); (**b**) reduced size and a distorted irregular surface with discrete protrusions (P) and leakage (L) of internal contents; (**c**) distortion of apical end and irregular surface with erosions (E) and protrusions (P); (**d**) reduced size with compression (C) and surface erosions (E); (**e**) compression (C) at the apical end, cracked (Cr) and eroded (E) surface with protrusions (P); (**f**) surface irregularities and protrusions (P) with leakage (L) of internal contents; (**g**) swollen bodies with surface erosions (E) and leakage (L) of internal contents; (**h**) a surface dimple (D) and prominent bulbous protrusion (P); (**i**) burst end (B), protrusion (P) and leakage (L) of internal contents

In IC mice, tachyzoites collected from INF–NT mice (subgroup Ia3) were generally crescent-shaped, average-sized, with completely smooth and regular surfaces (Fig. 6 a). On the other hand, tachyzoites retrieved from the INF–T mice (subgroup Ib) showed obvious ultrastructural alterations. Some tachyzoites were reduced in size (Fig. 6 b and d) with distorted irregular surfaces (Fig. 6 b, c, e, f, and g), while others were compressed (Fig. 6 d and e) or swollen (Fig. 6 g). Moreover, in most tachyzoites, discrete surface protrusions (Fig. 6 b, c, e, f, h, and i), erosions (Fig. 6 c, d, e, and g), cracks (Fig. 6 e), or dimples (Fig. 6 h) were revealed. In addition, leakage of internal contents was noticed in some tachyzoites (Fig. 6 b, f, g, and i). Furthermore, a burst was observed in others (Fig. 6 i).

**Fig. 7:**
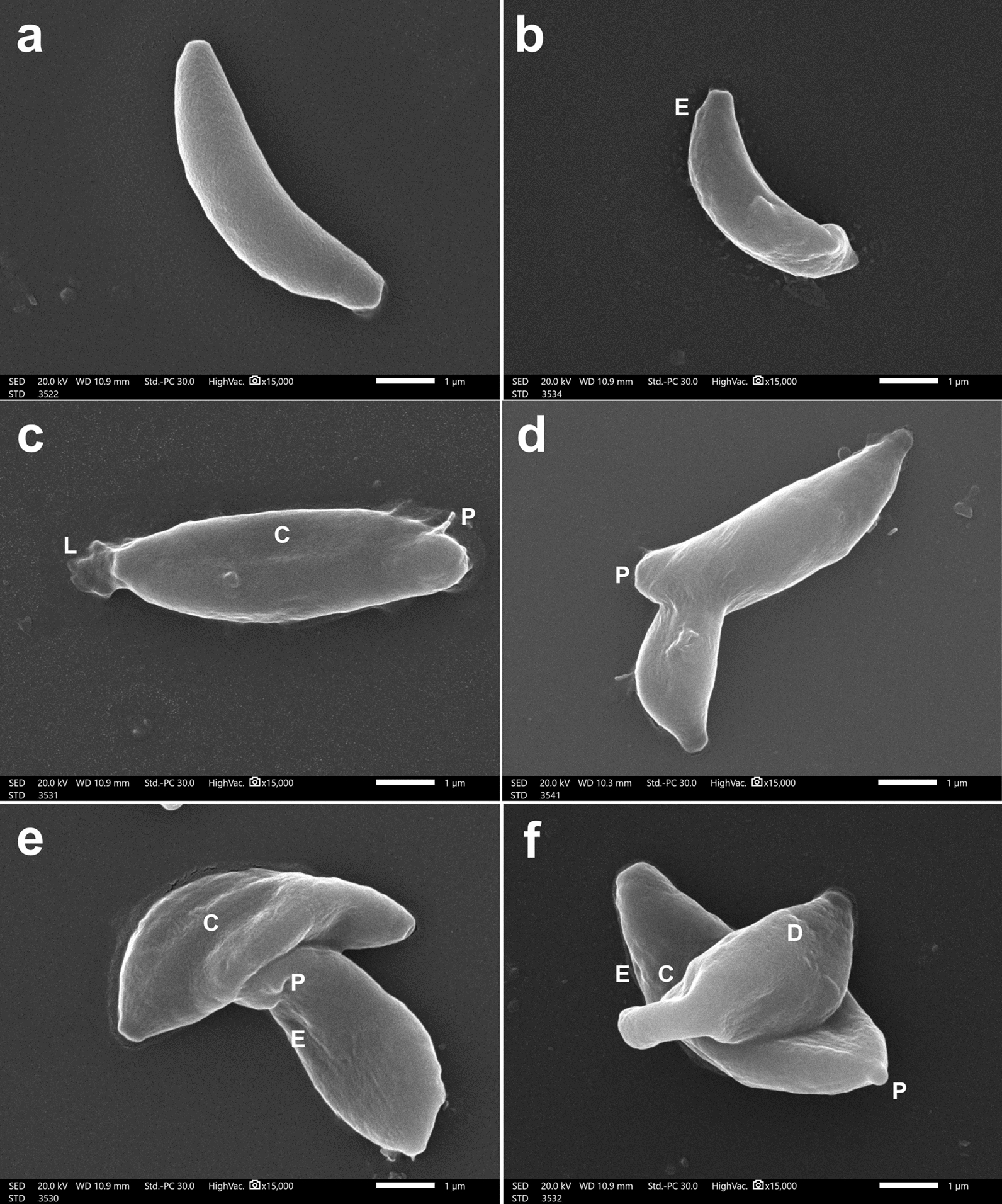
SEM of *T. gondii* tachyzoites retrieved from the peritoneal fluid of INF IS mice (×15,000): (**a**) NT tachyzoite (subgroup IIa3) showing a crescent shape with a completely smooth surface. (**b**–**f**) CCV-T tachyzoites (subgroup IIb); (**b**) reduced size and irregular surface with erosion (E); (**c**) disfigurement of the apical region, with leakage (L) of internal contents, surface compression (C) and protrusions (P); (**d**) disfigured ends, twisting and prominent protrusion (P); (**e**) twisting and compression (C) with surface protrusions (P) and erosions (E); (**f**) swollen bodies and compression (C) with surface protrusion (P), erosions (E) and dimples (D)

In IS mice, tachyzoites harvested from INF–NT mice (subgroup IIa3) were typically normal (Fig. 7 a). Contrarily, tachyzoites retrieved from the INF–T mice (subgroup IIb) exhibited notable ultrastructural alterations. Some tachyzoites displayed size reduction (Fig. 7 b). Most tachyzoites were disfigured with altered surfaces, erosions (Fig. 7 b, e, and f), compression (Fig. 7 c, e, and f), or twisted (Fig. 7 d and e). In addition, some tachyzoites possessed disfigured ends (Fig. 7 c and d), discrete protrusions (Fig. 7 c, d, e, and f), or dimples (Fig. 7 f). Leakage of internal contents was detected in some tachyzoites (Fig. 7 c).

#### TEM

**Fig. 8:**
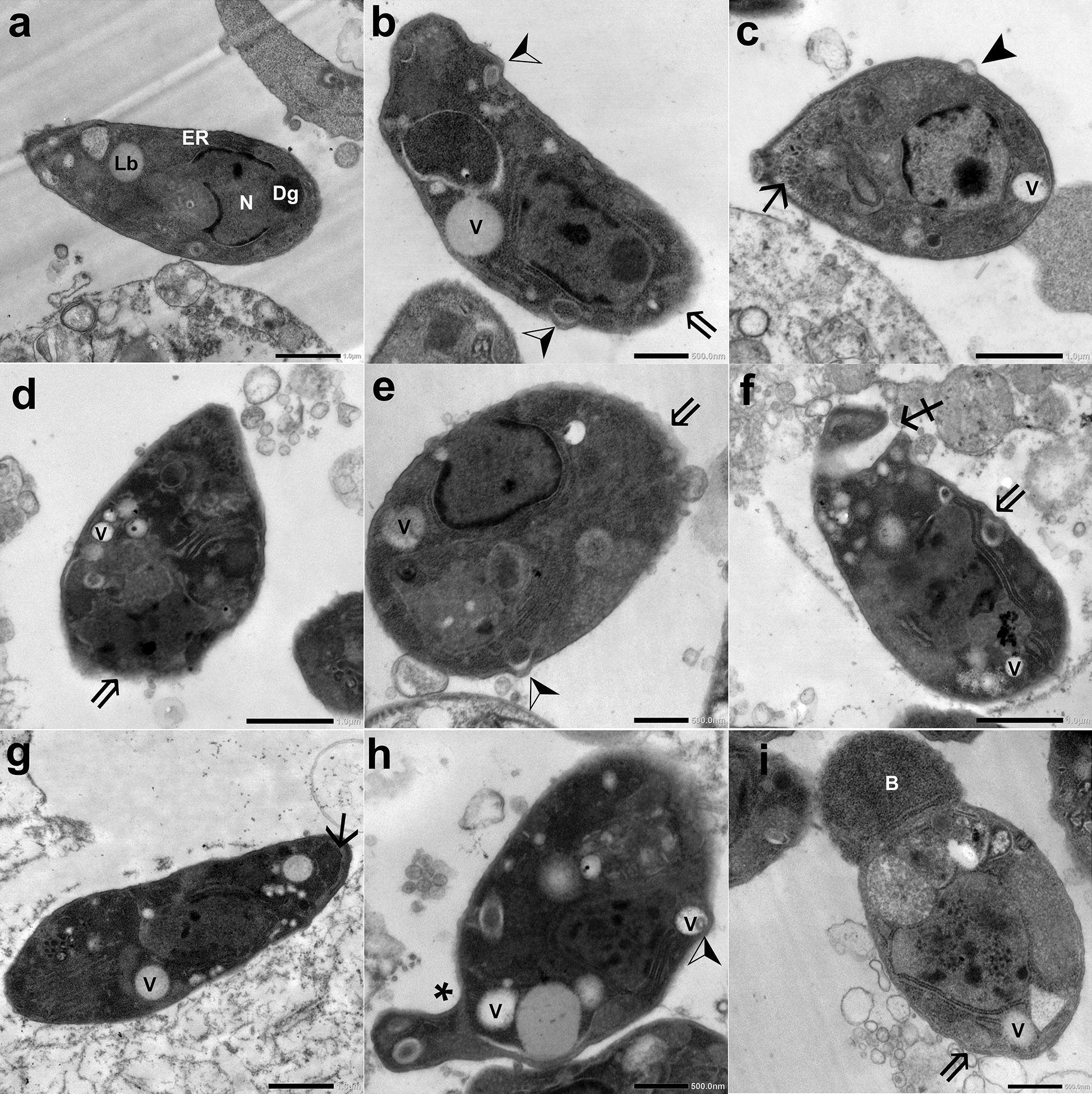
TEM of *T. gondii* tachyzoites retrieved from the peritoneal fluid of INF IC mice: (**a**) NT tachyzoite (subgroup Ia3) and (**b**–**i**) CCV-T tachyzoites (subgroup Ib) showing (Lb) lipid body, (Dg) dense granules, (N) nucleus, (ER) endoplasmic reticulum, ($$\Rightarrow $$) disrupted plasma membrane, (V) cytoplasmic vacuole, (➢) protrusions, ($$\searrow$$) separation of plasma membrane, (▶) surface bleb, (♐) dissociation of the apical end; ($$* $$) constriction, (B) burst apical end. **a**, **c**, **d**, **f**, and **g**: (×10,000); **b**, **e**, **h**, and **i**: (×5000)

In IC mice, extracellular tachyzoites collected from the peritoneal fluid of INF–NT mice (subgroup Ia3) were crescent-shaped and average-sized with lipid bodies, endoplasmic reticulum, dense granules, and an intact regular plasma membrane enclosing the nucleus with an intact membrane (Fig. 8 a). On the other hand, tachyzoites retrieved from the INF–T mice (subgroup Ib) showed evident ultrastructural alterations. Some tachyzoites were swollen (Fig. 8 c–e), while others were disfigured (Fig. 8 h and i). The plasma membranes of most of them were disrupted (Fig. 8b, d, e, f and i) or separated (Fig. 8 c and g). Protrusions (Fig. 8 b, e, and h) and blebs (Fig. 8 c) were noticed in some tachyzoites. Other tachyzoites possessed constriction (Fig. 8 h) or burst (Fig. 8 i). In addition, the apical ends of some tachyzoites were dissociated (Fig. 8 f) or distorted (Fig. 8 h). The cytoplasm of most tachyzoites was vacuolated (Fig. 8 b–i), while in some it was disintegrated (Fig. 8 d). Furthermore, nuclear fragmentation (Fig. 8 f, h, and i) was observed in some tachyzoites.

**Fig. 9:**
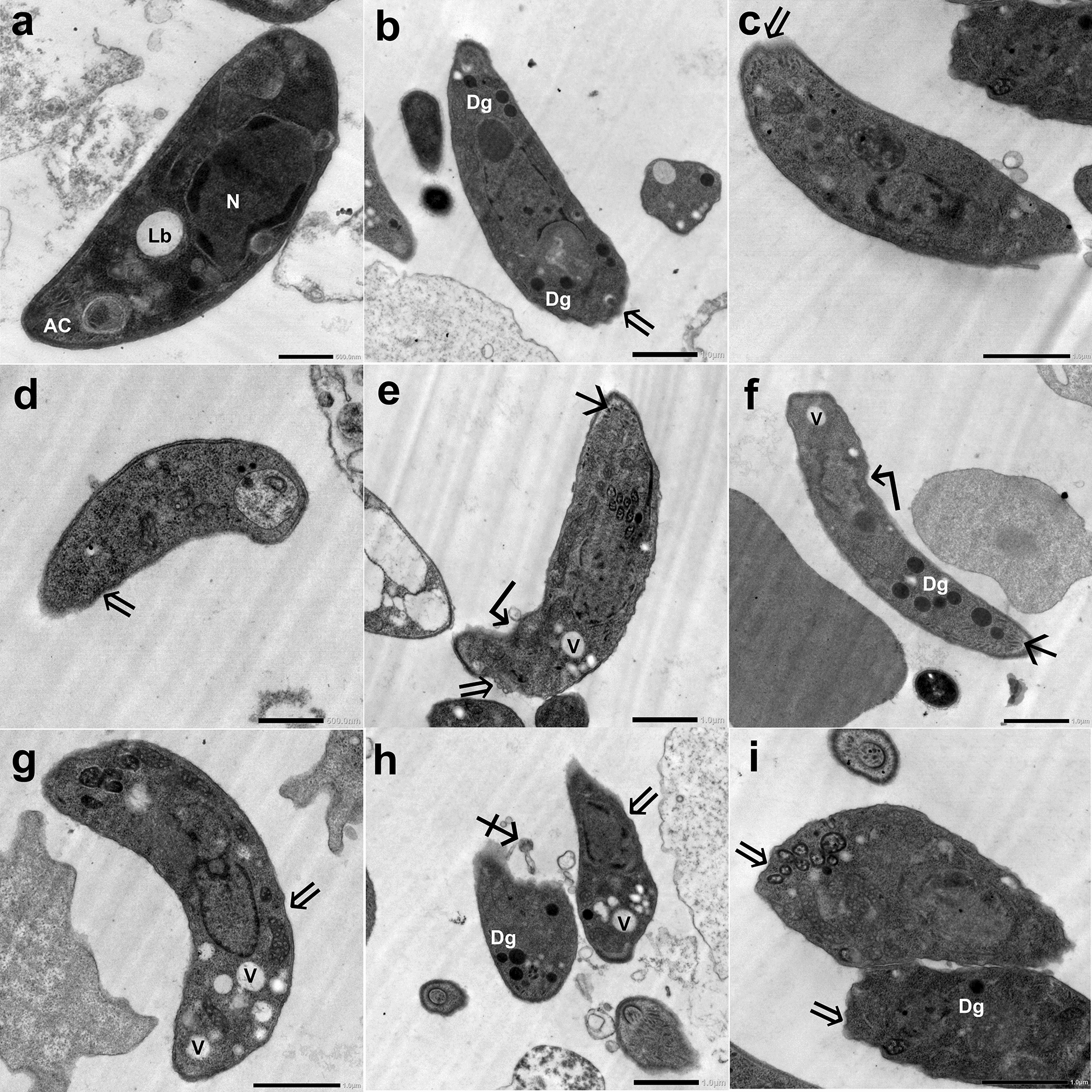
TEM of *T. gondii* tachyzoites retrieved from the peritoneal fluid of INF IS mice: (**a**) NT tachyzoite (subgroup IIa3) and (**b**–**i**) CCV-T tachyzoites (subgroup IIb). AC, apical complex; Lb, lipid body; N, nucleus; Dg, dense granules; $\Rightarrow$, irregular plasma membrane; $\hookleftarrow$, indentation; V, cytoplasmic vacuole; $\searrow$, separated plasma membrane; ♐, dissociation of the apical end. **a** and **d**: (×5000); **b**, **c** and **e**–**i**: (×10,000)

In IS mice, tachyzoites collected from the INF–NT mice (subgroup IIa3) were typically normal (Fig. 9 a). However, tachyzoites collected from the INF–T mice (subgroup IIb) were altered. Some tachyzoites were reduced in size and distorted (Fig. 9 h), while others were swollen (Fig. 9 i). The plasma membranes of most of them were irregular and disrupted (Fig. 9 b–e and g–i). Indentations and separation of plasma membranes were noticed in some tachyzoites (Fig. 9 e and f). While dissociation of apical ends was observed in others (Fig. 9 h). Additionally, many cytoplasmic vacuoles (Fig. 9 e, f, g, and h) and dense granules (Fig. 9 b, f, h, and i) were noticed in some tachyzoites. Moreover, others revealed nuclear fragmentation (Fig. 9 c and e) or disintegration of their internal contents (Fig. 9 d and h).

**Fig. 10:**
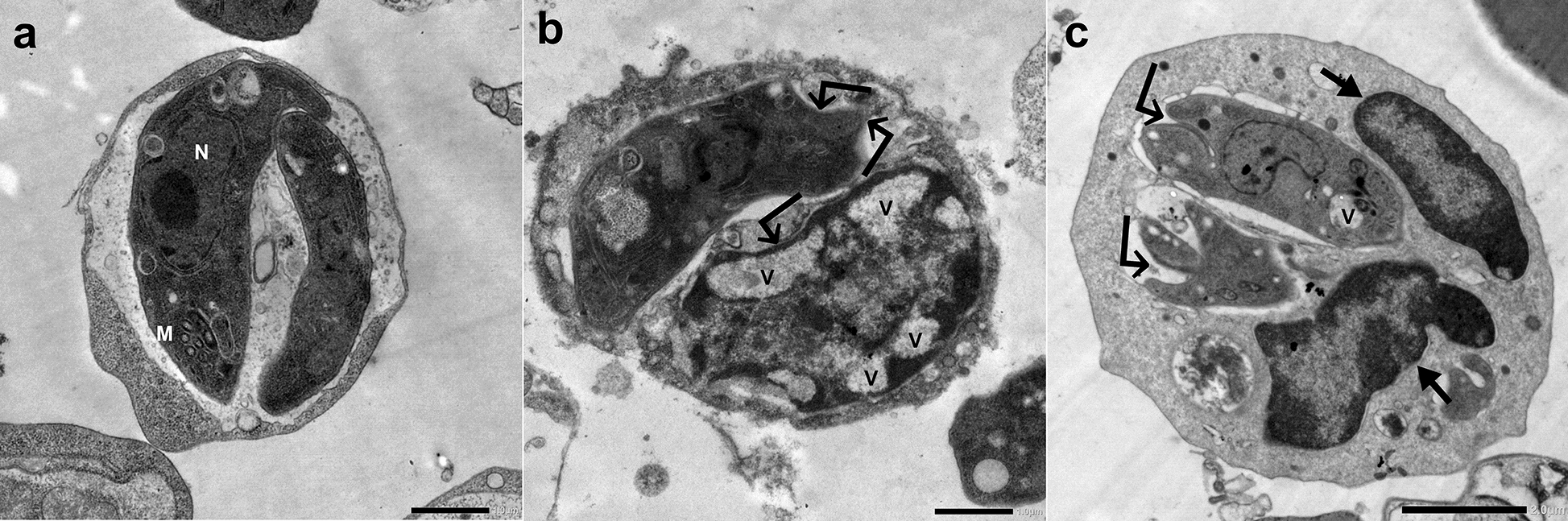
TEM of *T. gondii* tachyzoites in the macrophage retrieved from the peritoneal fluid of INF subgroups: (**a**) NT, (**b**) CCV-T IC, and (**c**) CCV-T IS mice; N, tachyzoite’s nucleus; M, mitochondria; →, macrophage’s nucleus; $\hookleftarrow$, indentation; V, cytoplasmic vacuole. **a** and **b** (×10,000); **c** (×20,000)

In addition, tachyzoites in macrophages retrieved from the peritoneal fluid of INF–NT mice (subgroup Ia3) were generally crescent-shaped with intact, regular plasma membranes and nuclear membranes. On the other hand, tachyzoites in macrophages obtained from either INF–T IC or IS mice (subgroups Ib and IIb, respectively) were distorted, with indentations, cytoplasmic vacuolations, nuclear fragmentation, or even complete disintegration of their internal contents (Fig. 10).

### Biochemical study

#### Liver function markers

**Fig. 11:**
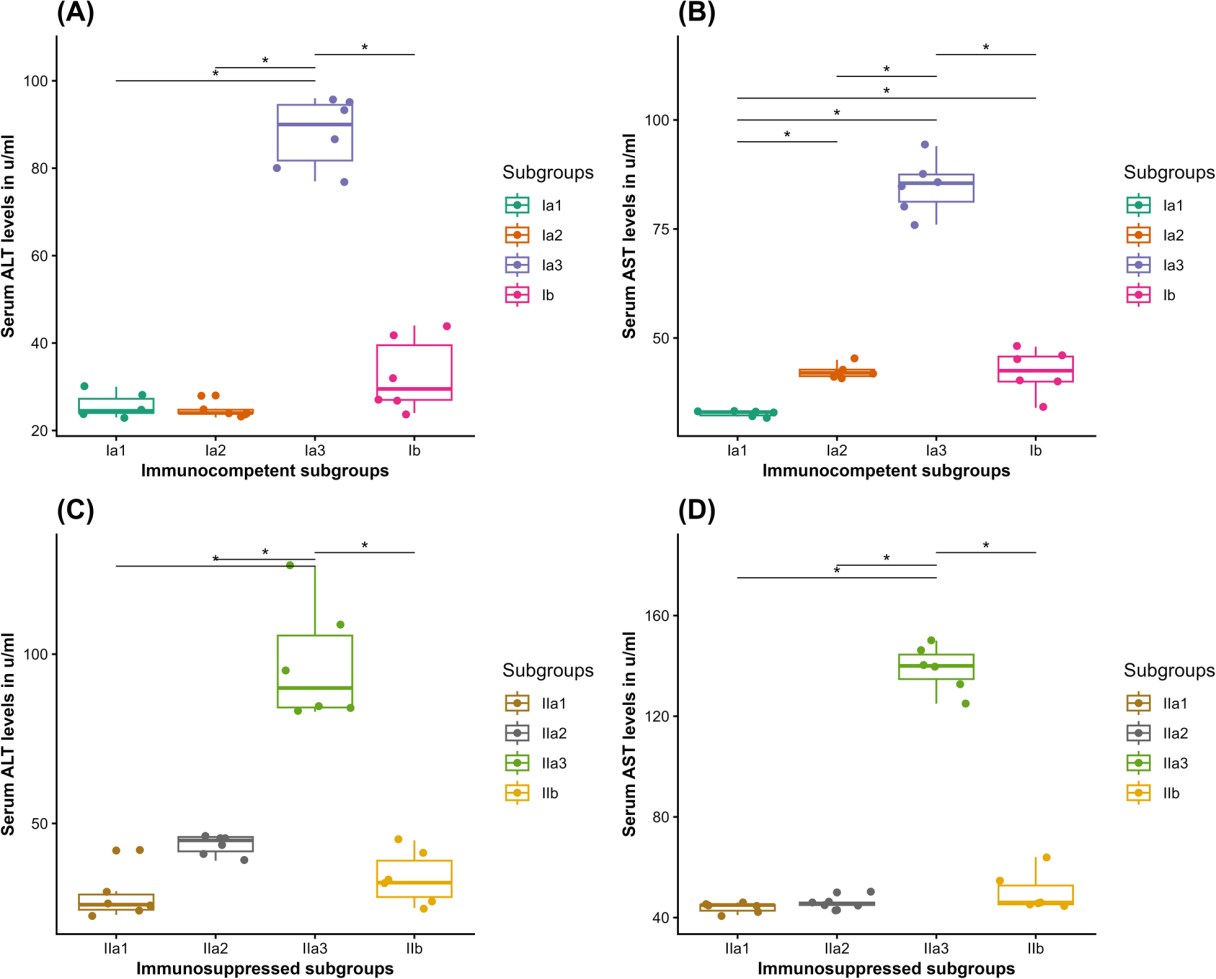
Boxplots demonstrating the liver function markers (AST and ALT) in U/ml in serum of the studied subgroups: (**A**) ALT in IC mice; (**B**) AST in IC mice; (**C**) ALT in IS mice; (**D**) AST in IS mice. Ia1, NI–NT IC subgroup; Ia2, NI–T IC subgroup; Ia3, INF–NT IC subgroup; Ib, INF–T IC subgroup; IIa1, NI–NT IS subgroup; IIa2, NI–T IS subgroup; IIa3, INF–NT IS subgroup; and IIb, INF–T IS subgroup. *Statistically significant at *P* < 0.05 alpha level

In IC mice, the mean ALT level was statistically insignificantly lower in the NI–T subgroup (Ia2) (24.67 ± 1.8 U/ml) compared with the NI–NT subgroup (Ia1) (25.67 ± 2.7 U/ml) (Wilcoxon rank sum test, *Z* = 21.5, *P* = 1). The mean AST level was statistically significantly higher in subgroup Ia2 (42.33 ± 1.5 U/ml) compared to subgroup Ia1 (32.67 ± 0.5 U/ml) (Wilcoxon rank sum test, *Z* = 0, *P* = 0.025). Meanwhile, the INF–NT subgroup (Ia3) showed statistically significantly elevated ALT (88.00 ± 8 U/ml) and AST (84.83 ± 6.3 U/ml) levels compared with both Ia1 and Ia2 subgroups (Wilcoxon rank sum test, [*Z* = 0, *P* = 0.03, 0.026] and [*Z* = 0, *P* = 0.029, 0.03], respectively). On the other hand, the INF–T subgroup (Ib) recorded statistically significantly lower ALT (32.67 ± 8.4 U/ml) and AST (42.17 ± 5.2 U/ml) levels compared with the subgroup (Ia3) (Wilcoxon rank sum test, *Z* = 36, *P* = 0.03, each) (Fig. 11 A and B).

In IS mice, the NI–T subgroup (IIa2) showed statistically insignificant higher ALT (43.67 ± 3.0 U/ml) and AST (45.83 ± 2.3 U/ml) levels compared with the NI–NT subgroup (IIa1) (28.5 ± 7.0 U/ml and 44.00 ± 2.0 U/ml, respectively) (Wilcoxon rank sum test, [*Z* = 2, *P* = 0.074], [*Z* = 10, *P* = 1]). On the other hand, statistically significantly higher ALT (97.00 ± 17.3 U/ml) and AST (139.0 ± 9.0 U/ml) levels were recorded in the INF–NT subgroup IIa3 compared with both IIa1 and IIa2 subgroups (Wilcoxon rank sum test, [*Z* = 0, *P* = 0.03, 0.028] and [*Z* = 0, *P* = 0.029, each], respectively). Noticeably, treatment in the INF–T subgroup (IIb) led to a statistically significant reduction in ALT (33.83 ± 7.8 U/ml) and AST (50.17 ± 7.8 U/ml) levels compared with subgroup IIa3 (Wilcoxon rank sum test, [*Z* = 36, *P* = 0.013], [*Z* = 36, *P* = 0.029], respectively) (Fig. 11 C and D).

#### Kidney function markers

**Fig. 12:**
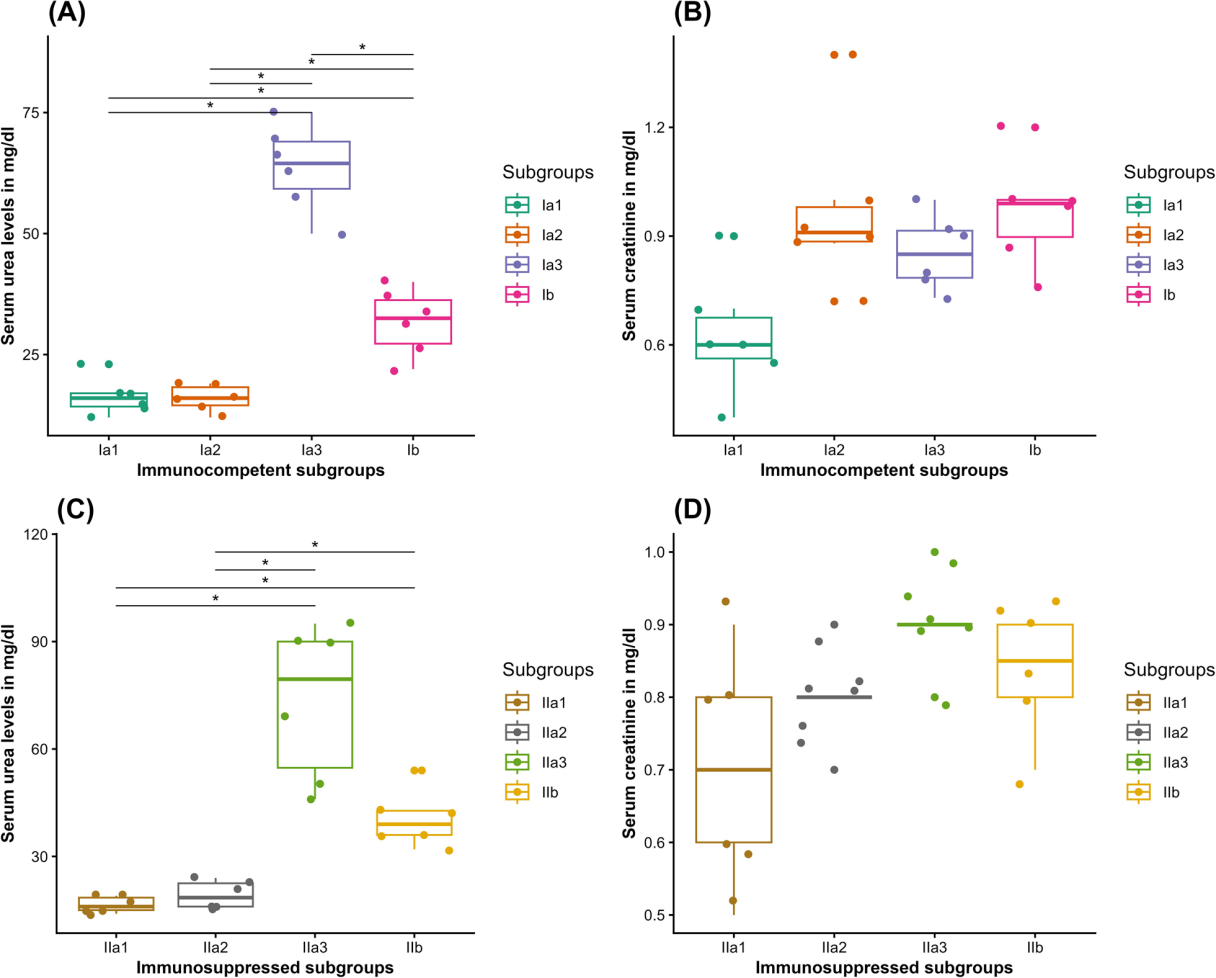
Boxplots demonstrating the kidney function markers in mg/dl in serum of the studied subgroups: (**A**) urea in IC mice; (**B**) creatinine in IC mice; (**C**) urea in IS mice; (**D**) creatinine in IS mice. Ia1, NI–NT IC subgroup; Ia2, NI–T IC subgroup; Ia3, INF–NT IC subgroup; Ib, INF–T IC subgroup; IIa1, NI–NT IS subgroup; IIa2, NI–T IS subgroup; IIa3, INF–NT IS subgroup; and IIb, INF–T IS subgroup. *Statistically significant at *P* < 0.05 alpha level

In IC mice, there was a statistically insignificant difference in the mean serum urea between subgroups Ia1 and Ia2 (Wilcoxon rank sum test, *Z* = 18, *P* = 1). Meanwhile, it was statistically significantly higher in subgroup Ia3 (63.67 ± 8.9 mg/dl) compared with subgroups Ia1 and Ia2 (16.33 ± 3.8 mg/dl and 16.00 ± 2.8 mg/dl, respectively) (Wilcoxon rank sum test, [*Z* = 0, *P* = 0.03], each). Noticeably, a statistically significant reduction in the mean serum urea level was observed in subgroup Ib (31.67 ± 6.8 mg/dl) compared with subgroup Ia3 (Wilcoxon rank sum test, *Z* = 36, *P* = 0.013) (Fig. 12 A).

Concerning the IS mice, there was a statistically insignificant increase in the mean serum urea in subgroup IIa2 compared to subgroup IIa1 (Wilcoxon rank sum test, *Z* = 10, *P* = 1). However, it was statistically significantly higher in subgroup IIa3 (73.33 ± 21.6 mg/dl) compared with subgroup IIa1 (16.5 ± 2.2 mg/dl) and subgroup IIa2 (19.17 ± 4.0 mg/dl) (Wilcoxon rank sum test, [*Z* = 0, *P* = 0.029], [*Z* = 0, *P* = 0.03], respectively). Treatment in the infected subgroup IIb resulted in a statistically insignificant reduction in the mean serum urea level (40.50 ± 7.8 mg/dl) compared to subgroup IIa3 (Wilcoxon rank sum test, *Z* = 34, *P* = 0.076) (Fig. 12 C).

On the other hand, the serum level of creatinine was within the normal range in all studied subgroups, regardless of infection, whether IC or IS, with no statistically significant difference compared with the corresponding subgroups (Fig. 12 B and D).

#### Oxidative stress markers

**Fig. 13:**
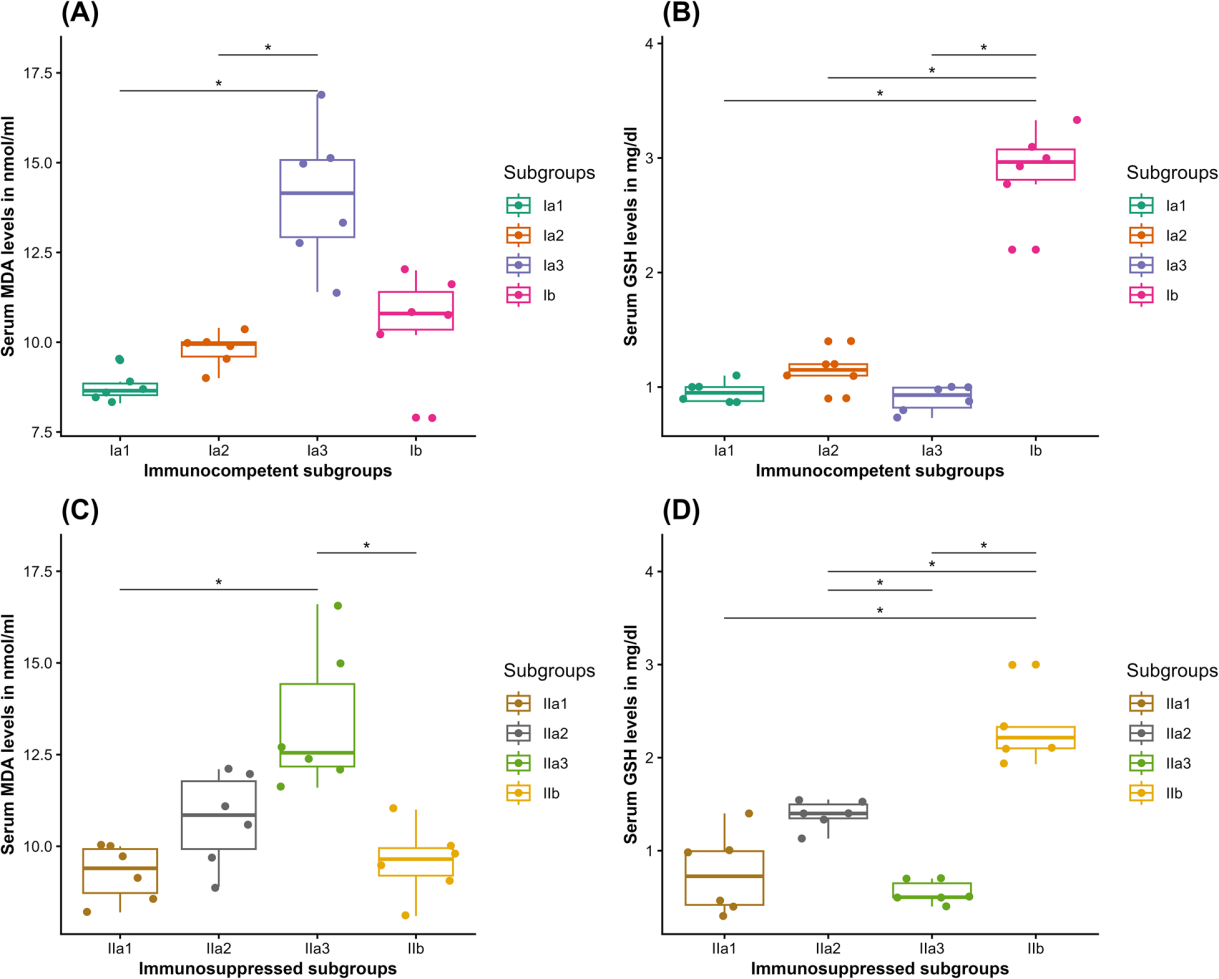
Boxplots demonstrating the oxidative stress markers (MDA in nmol/ml and GSH in mg/dl) in serum of the studied subgroups: (**A**) MDA in IC mice; (**B**) GSH in IC mice; (**C**) MDA in IS mice; (**D**) GSH in IS mice. Ia1, NI–NT IC subgroup; Ia2, NI–T IC subgroup; Ia3, INF–NT IC subgroup; Ib, INF–T IC subgroup; IIa1, NI–NT IS subgroup; IIa2, NI–T IS subgroup; IIa3, INF–NT IS subgroup; and IIb, INF–T IS subgroup. *Statistically significant at *P* < 0.05 alpha level

In IC mice, a statistically insignificant increase in the mean MDA level was noticed in subgroup Ia2 (9.80 ± 0.5 nmol/ml) compared to subgroup Ia1 (8.750 ± 0.4 nmol/ml) (Wilcoxon rank sum test, *Z* = 1.5, *P* = 0.061). The mean MDA level was statistically significantly higher in subgroup Ia3 (14.08 ± 2.0 nmol/ml) compared with subgroups Ia1 and Ia2 (Wilcoxon rank sum test, [*Z* = 0, *P* = 0.013], [*Z* = 0, *P* = 0.03], respectively). Otherwise, treatment in the INF–T subgroup Ib induced a statistically insignificant reduction in the mean MDA (10.55 ± 1.4 nmol/ml) compared with subgroup Ia3 (Wilcoxon rank sum test, [*Z* = 34, *P* = 0.077). Moreover, it was statistically insignificant higher in subgroup Ib compared with subgroup Ia2 (Wilcoxon rank sum test, *Z* = 7, *P* = 0.55). Concerning GSH, there was a statistically insignificant difference among the three CTL subgroups Ia1, Ia2, and Ia3 (0.957 ± 0.1, 1.15 ± 0.2, and 0.898 ± 0.1 mg/dl, respectively) (Wilcoxon rank sum test, [*Z* = 4.5, *P* = 0.208], [*Z* = 23, *P* = 1], and [*Z* = 33, *P* = 0.118]). Nevertheless, the INF–T subgroup Ib showed a statistically significantly higher mean GSH level (2.888 ± 0.4 mg/dl) compared with subgroup Ia3 (Wilcoxon rank sum test, *Z* = 0, *P* = 0.03) (Fig. 13 A and B).

Regarding the IS mice, a statistically insignificant increase in the mean MDA level was observed in subgroup IIa2 (10.73 ± 1.3 nmol/ml) compared with subgroup IIa1 (9.267 ± 0.8 nmol/ml) (Wilcoxon rank sum test, *Z* = 6.5, *P* = 0.463). Whereas the mean MDA level in subgroup IIa3 (13.40 ± 2.0 nmol/ml) was statistically significantly higher compared with subgroup IIa1 (Wilcoxon rank sum test, *Z* = 0, *P* = 0.03) and statistically insignificantly higher compared with subgroup IIa2 (Wilcoxon rank sum test, *Z* = 2.5, *P* = 0.097). On the other hand, treatment of the INF–T subgroup IIb resulted in a statistically significant decrease in the mean MDA level (9.583 ± 1.0 nmol/ml) compared with subgroup IIa3 (Wilcoxon rank sum test, *Z* = 36, *P* = 0.013). Concerning the mean GSH level, it was statistically insignificantly higher in subgroup IIa2 (1.390 ± 0.2 mg/dl) compared with subgroup IIa1 (0.758 ± 0.4 mg/dl) (Wilcoxon rank sum test, *Z* = 3, *P* = 0.116). While it was statistically insignificantly lower in subgroup IIa3 (0.55 ± 0.1 mg/dl) compared with subgroup IIa1 (Wilcoxon rank sum test, *Z* = 19.5, *P* = 1) and statistically significantly lower compared with subgroup IIa2 (Wilcoxon rank sum test, *Z* = 36, *P* = 0.028). Moreover, the mean GSH level in the experimental subgroup IIb (2.298 ± 0.4 mg/dl) was statistically significantly higher compared with all three CTL subgroups, IIa1, IIa2, and IIa3 (Wilcoxon rank sum test, [*Z* = 0, *P* = 0.03], [*Z* = 0, *P* = 0.029], [*Z* = 0, *P* = 0.027], respectively) (Fig. 13 C and D).

## Discussion

The control of human *T. gondii* infection depends primarily on conventional chemotherapeutic agents, which pose a significant challenge [2]. To address serious concerns, the anti-*Toxoplasma* activity of a sublethal dose of CCV in murine models has been investigated for the first time as a safe and effective natural therapeutic alternative.

Concerning the INF–NT IC and IS subgroups, a decrease in food intake and lethargic behavior was observed, and 8.3% and 16.7% of mice died before the day of sacrifice, respectively, with a significant reduction in their mean survival time. This could be related to the virulence of RH strain, leading to a high parasite burden, which induced their death [36]. Higher MR and more pronounced behavioral alterations were reported in IS mice due to the additional effect of cyclophosphamide [37]. On the contrary, normal behavior with no deaths and prolonged survival of the NI–T mice, especially the IC, indicated that the used dose of CCV was well-tolerated with a favorable safety profile. Furthermore, treatment of the INF–T mice with one-fourth LD50 of CCV (LD = 0.535 mg/kg) improved their behavior and food intake, referring to its ability to ameliorate the impact of infection. Similar results were reported in a previous study after IP injection of ^1^/_10_ LD50 of CCV (LD50 = 2.4 mg/kg) in mice [20]. Moreover, a statistically significant increase in their mean survival time was recorded, which could be attributed to the protective effect of CCV that hindered the invasion and intracellular multiplication of tachyzoites, hence giving them a substantially longer survival time [38].

Conspicuously, there was a statistically significant reduction in the peritoneal and hepatic parasite load among INF–T IC and IS subgroups compared with their corresponding INF CTL, indicating that the used sublethal dose of CCV exhibited an astounding anti-*Toxoplasma* effect. This significant activity could be attributed to its bioactive components, especially PLA2s, LAAOs, and MPs. Concerning PLA2s, they are known to stimulate immune response against the parasite and interfere with tachyzoites’ membrane, reducing their invasion into the host cell [39]. Other components have been engaged as LAAOs that possess direct action on the cell plasma membrane by producing hydrogen peroxide and partially depleting essential amino acids from the cell. While MPs were reported to degrade extracellular matrix components and prevent *T. gondii* tachyzoites from invasion of nearby host cells and ultimately, their dissemination. [40, 41]. For instance, Allane et al. reported that treatment with the disintegrin component isolated from CCV inhibited the growth of *L. infantum* promastigotes by 84.7% [42]. Recent studies have highlighted the potential anti-schistosomal activity of CCV in a time and dose-dependent manner, both in vitro and in vivo [16, 22].

Regarding the animal infectivity, there was a statistically significant decrease in parasite loads in the peritoneal fluid and liver tissues of INF–T IC and IS subgroups compared with their corresponding INF CTLs. The proteolytic activity of MPs impairs the parasitophorous vacuole’s functional activity and the formation of the daughter cells’ plasmalemma, thereby reducing their invasion and intracellular replication, which might be the possible explanation for the decrease in their infectivity and number [41]. Moreover, Izidoro et al. had demonstrated that treatment of *T. gondii* tachyzoites by LAAOs isolated from *Bothrops pirajai* venom in vitro exhibited a dose-dependent inhibitory effect on the parasite's infection index, depending on the time of administration. They proposed that LAAOs caused oxidative alterations in the extracellular matrix of fibroblasts that hindered the parasite’s ability to utilize laminin and reach host cell membrane receptors, thus reducing fibroblast infection rates [40].

The ultrastructural study substantiated the parasitological and molecular results. SEM examination of *T. gondii* tachyzoites collected from the peritoneal fluid of the INF–T IC and IS mice revealed evident topographic alterations. This was supported by an in vivo study reporting ultrastructural tegumental alterations of *S. mansoni* adult worms treated with CVV in a dose-dependent manner [16]. Parallelly, TEM examination of the tachyzoites, either free or in macrophages retrieved from the INF–T subgroups, demonstrated the detrimental effect of CCV on them. The multiple cytoplasmic vacuoles noted might have been induced by PLA2s that degraded the phospholipids in the tachyzoites’ membranes, compromising their integrity, fluidity, permeability, and permitting the entry of extracellular fluids [43]. In the same vein, some tachyzoites lost their cytoplasmic and nuclear membrane integrity, and nuclear fragmentation was observed [39]. Consistent with another study, we postulated that protrusions, blebs, and even complete disintegration of the internal content of some treated tachyzoites could be mediated by zinc and cationic peptides found in MPs that had displaced cellular metal ions, damaged the outer membrane, and resulted in cell disintegration [44]. The ultrastructural alterations could explain the reduction in invasion, intracellular proliferation, parasite burden, and decline in the infectivity of the CCV-treated tachyzoites.

The liver function markers, AST and ALT, act as sensitive indicators for identifying hepatocellular injury and loss of cell membrane functional integrity [20]. A statistically significant elevation in serum liver transaminase levels was observed in both INF–NT IC and IS subgroups, highlighting the rapid and extensive proliferation of *T. gondii,* resulting in focal necrosis of liver cells [45]. Moreover, the higher levels of serum liver markers observed in the INF–NT IS mice could be attributed to temporary hepatotoxicity or infection flare-up linked to cyclophosphamide treatment [46, 47]. On the other hand, CCV treatment of the INF–T mice induced a statistically significant reduction in the mean values of serum liver markers in both IC and IS subgroups, which might be explained by its anti-*Toxoplasma* effects, as proven by the decrease in parasite burden, thereby diminishing the anticipated impact of infection on the liver. Furthermore, the insignificant changes in the mean values of serum liver function markers recorded in the NI–T IC and IS subgroups could be attributed to the nonsignificant effect of the low dose of CCV on the liver cells and their enzymes [20].

The renal function markers, urea and creatinine, are reliable indices for identifying renal damage and impairment of its functional capability to filter blood [33]. A statistically significant increase in serum urea was observed in the INF–NT IC and IS subgroups could be attributed to the detrimental effect of the virulent *T. gondii* strain on the kidneys [48]. Consistently, accumulated evidence pointed to the deleterious impact of infection on the kidneys [45, 49]. Meanwhile, CCV treatment of the INF–T mice induced a decrease in mean serum urea levels, which could be attributed to the reduction of the parasite burden and subsequent alleviation of the negative impact of infection on the kidneys. The decrease in serum urea in response to various doses of CCV was reported in several studies [20, 21, 50].^.^ In addition, mean values of urea in the NI–T subgroups were roughly similar to those in the NI–NT CTL, indicating the safety of the used low dose of CCV on the kidney. On the other hand, the serum level of creatinine was within the normal range in all studied subgroups, whether infected or not, IC or IS. Similar results were reported by Da Silva et al.; a nonsignificant difference in creatinine levels was detected despite the urea level being significantly higher in INF mice than NI CTL mice [47]. It is plausible that the mean kidney function markers in the IS subgroups were approximately equivalent to their corresponding IC subgroups, indicating that oral cyclophosphamide was well tolerated by the kidneys [51].

The oxidative stress markers are indicators used to evaluate the degree of oxidative damage [52]. Concerning the infection-induced oxidative stress, a statistically significant elevation in MDA and a statistically insignificant reduction in GSH in sera of the INF–NT IC and IS subgroups were detected. This could be attributed to the infection-mediated lipid peroxidation in the cell membrane [53]. In parallel to the current results, the serum MDA level was markedly enhanced with a decrease in the GSH level in mice infected with *T. gondii* in previous studies [54, 55]. On the contrary, in the INF–T IC and IS subgroups, MDA was noticeably decreased, and GSH increased, indicating that the high antioxidant activity of the used low dose of CCV could alleviate the oxidative injuries induced by the infection. This might be ascribed to the effect of PLA2s present in CCV that potentially served as an antioxidant suppressing the formation of reactive oxygen species [50, 56].

A head-to-head comparison demonstrated a slightly higher anti-*Toxoplasma* activity of the used sublethal dose of CCV (one-fourth LD50) in the IC animals than in the IS ones through molecular, parasitological, and ultrastructural studies. This could be referred to the combined effect of the CCV and the host’s immune response on the tachyzoites in IC subgroups. The good tolerability of this dose of CCV in both IC and IS mice was evidenced by the almost comparable results displayed in MR, survival percentage, and biochemical study. Demonstrating good tolerability in IS mice suggested that the used sublethal dose of CCV was less likely to cause serious complications, which is a major challenge for any treatment in this group and offers hope for a safe and potentially effective agent despite the compromised immune status.

## Conclusions

The current study is the first evidence on the effectiveness and safety of a sublethal dose of CCV as a proficient therapeutic agent against acute toxoplasmosis in both IC and IS mice. Besides its high antioxidant activity, it reduced parasite burden, animal infectivity with spectacular topographic alterations. Thus, one-fourth LD50 of crude CCV is a promising, effective, natural alternative to the conventional therapy for treating acute toxoplasmosis, particularly IS individuals who are unable to withstand the adverse side effects of the existing treatment. Building on our results, further studies are needed to investigate the anti-*Toxoplasma* activity of each major bioactive compound in CCV against RH and other virulent strains. Further research is required to study the histopathological changes induced by one-fourth LD50 of CCV across different organs and explore the underlying pharmacokinetics and dynamics. This study gave ground for investigating the potential activity of CCV against avirulent strains of *T. gondii*.

## Supplementary Information

Additional file 1. Pilot study

## Data Availability

All generated or analyzed relevant data are included in the manuscript or supplementary information file.

## Abbreviations

%R: Percent reduction
ALT: Alanine transaminase
AST: Aspartate transaminase
CCV: *Cerastes cerastes* venom
CRISPs: Cysteine-rich secretory proteins
Ct: Cycle threshold
CTL: Control
Dpi: Day post infection
GSH: Reduced glutathione
HEPES: 4-(2-Hydroxyethyl)-1-piperazineethanesulfonic acid
IC: Immunocompetent
INF–NT: Infected nontreated
INF–T: Infected treated
IP: Intraperitoneally
IR: Infectivity rate
IS: Immunosuppressed
LAAOs: L-amino acid oxidases
LD50: Lethal dose 50
Logs: Logarithms
MDA: Malondialdehyde
MPs: Metalloproteases
MR: Mortality rate
NI–NT: Noninfected nontreated
NI–T: Noninfected treated
OsO_4_: Osmium tetroxide
PLA2s: Phospholipases A2
qRT–PCR: Quantitative real-time polymerase chain reaction
SEM: Scanning electron microscopy
SPs: Serine proteases
*T. gondii*: *Toxoplasma gondii*
TEM: Transmission electron microscopy

## References

[1] Nayeri T, Moosazadeh M, Asl AD, Ghaffarifar F, Sarvi S, Daryani A. *Toxoplasma* infection and Rhesus blood group system: a systematic review and meta-analysis. PLoS ONE. 2023;18:e0287992. 37406027 10.1371/journal.pone.0287992 PMC10321609

[2] Khairullah AR, Kurniawan SC, Widodo A, Effendi MH, Hasib A, Silaen OSM, et al. A comprehensive review of toxoplasmosis: serious threat to human health. Open Public Health J. 2024;17: e18749445281387. 10.2174/0118749445281387240202094637.

[3] Garcia LS, Procop GW. Diagnostic medical parasitology. 6th ed. Washington: ASM Press; 2016.

[4] Guglielmi P, Secci D. Treatment of toxoplasmosis: An insight on epigenetic drugs. In: Antiprotozoal drug development and delivery. Cham: Springer; 2022.

[5] Portnoy JM, Pandya A. Drug Allergy. In: Pandya A, editor. Textbook of allergy for the clinician. Boca Raton: CRC Press; 2021.

[6] Ben-Harari RR, Goodwin E, Casoy J. Adverse event profile of pyrimethamine-based therapy in toxoplasmosis: a systematic review. Drugs R D. 2017;17:523–44. 28879584 10.1007/s40268-017-0206-8 PMC5694419

[7] (WHO) WHO Geneva. Traditional medicine strategy. 2002–2005;1–59.

[8] Suárez VEY, Sepúlveda GDP, Contreras FA, Capaldo AA, Núñez JJ, Trovatti E. Rheological and biological properties of adhesive skin secretions from *Eupsophus vertebralis* (Anura: Alsodidae). 2024. 10.1155/2024/2722351 PMC10985274 38566624

[9] Silva MF, Mota CM, Miranda VS, Cunha AO, Silva MC, Naves KS, et al. Biological and enzymatic characterization of proteases from crude venom of the ant *Odontomachus bauri*. Toxins. 2015;7:5114–28. 26633501 10.3390/toxins7124869 PMC4690119

[10] De Assis DRR, Pimentel PMO, Dos Reis PVM, Rabelo RAN, Vitor RWA, Cordeiro MDN, et al. *Tityus serrulatus* (scorpion): From the crude venom to the construction of synthetic peptides and their possible therapeutic application against *Toxoplasma gondii* infection. Front Cell Infect Microbiol. 2021;11:706–18. 10.3389/fcimb.2021.706618 PMC8329421 34354963

[11] Gustavo TA, de Souza CMM, Oliveira PF, Motoie G, Mitsuyoshi HR, Maria AM, et al. Amphibian secretions for drug discovery studies: a search for new antiparasitic and antifungal compounds. Lett Drug Des Discov. 2007;4:67–73.

[12] Choi J-W, Lee J, Lee J-H, Park B-J, Lee EJ, Shin S, et al. Omega-3 polyunsaturated fatty acids prevent *Toxoplasma gondii* infection by inducing autophagy via AMPK activation. Nutrients. 2019;11:2137–53. 31500218 10.3390/nu11092137 PMC6771136

[13] Barakat AM, El-Razik KAA, El Fadaly HAM, Saleh WM, Ali FAZ, Gouda AA, et al. Parasitological, molecular, and histopathological investigation of the potential activity of propolis and wheat germ oil against acute toxoplasmosis in mice. Pharmaceutics. 2023;15:478. 36839800 10.3390/pharmaceutics15020478 PMC9967381

[14] Tanaka TOY, Saito A, Shimazaki K, Igarashi I, Suzuki N. Growth inhibitory effects of bovine lactoferrin to *Toxoplasma gondii* parasites in murine somatic cells. J Vet Med Sci. 1996;58:61–5. 8645758 10.1292/jvms.58.61

[15] Zona RDC, Aragón DM, Almeida AI. Innovations in snake venom-derived therapeutics: a systematic review of global patents and their pharmacological applications. Toxins (Basel). 2025;17:136. 40137909 10.3390/toxins17030136 PMC11945783

[16] Mahdy A, Mostafa OMS, Aboueldahab MM, Nigm AH. Antiparasitic activity of *Cerastes cerastes* venom on *Schistosoma mansoni* infected mice‏. Exp Parasitol. 2025;268:108866. 39617195 10.1016/j.exppara.2024.108866

[17] El Amir AM, Mohamed SG, Shaker LS, El Feky AA, Said W. Studies on venoms of the Egyptian cobra (*Naja haja*), the horned viper (*Cerastes cerastes*), and the honey bee (*Apis mellifera*): comparison safety study for 1/10 LD50. J Egypt Soc Parasitol. 2021;51:201–12.

[18] Soliman NS, Kandeil MA, Khalaf MM. *Cerastes* snake venom as a promising approach in the management of complete Freund’s adjuvant-induced rheumatoid arthritis in rats: involvement of RANKL and JAK/STAT pathway. J Ethnopharmacol. 2023;314:116577. 37178980 10.1016/j.jep.2023.116577

[19] Aziz M, Abdel-Rahman MA, Abbas O, Mohamed EA-M, Hassan M. Snake venoms-based compounds as potential anticancer prodrug: sand viper *Cerastes cerastes* as a model. Egypt J Chem. 2022;65:43–55.

[20] Aziz MM, Hassan MK, Abbas OA, Mohammed EA, Rahman MAA. Viper snake (*Cerastes cerastes*) venom overcomes the hepatocellular carcinoma in experimental rats: histological evidence. J Biotech Res. 2022;13:189–98.

[21] Shabaan A, El Feky A, Abdel Latif K, Moghib H. In vitro antibacterial and bio-histological effects of *Cerastes cerastes* venom on Albino mice. Biochem Lett. 2018;14:40–53.

[22] Hassan EA, Abdel-Rahman MA, Ibrahim MM, Soliman MF. In vitro antischistosomal activity of venom from the Egyptian snake *Cerastes cerastes*. Rev Soc Bras Med Trop. 2016;49:752–7. 28001223 10.1590/0037-8682-0241-2016

[23] Fernandez-Gomez R, Zerrouk H, Sebti F, Loyens M, Benslimane A, Ouaissi MA. Growth inhibition of *Trypanosoma cruzi* and *Leishmania donovani infantum* by different snake venoms: preliminary identification of proteins from *Cerastes cerastes* venom which interact with the parasites. Toxicon. 1994;32:875–82. 7985193 10.1016/0041-0101(94)90366-2

[24] El-Zawawy LA, El-Said D, Mossallam SF, Ramadan HS, Younis SS. Triclosan and triclosan-loaded liposomal nanoparticles in the treatment of acute experimental toxoplasmosis. Exp Parasitol. 2015;149:54–64. 25499511 10.1016/j.exppara.2014.12.007

[25] El-Zawawy LA, El-Said D, Hegazy IH, Shalaby TI, Arán VJ, Hezema NN. Effect of VAM2–2 and VAM2–2 loaded chitosan nanoparticles in treatment of experimental toxoplasmosis: an experimental study. Sens Sci. 2020;7:3.

[26] Silva L, Brandão G, Pinheiro B, Vitor R. Immunosuppression with cyclophosphamide favors reinfection with recombinant *Toxoplasma gondii* strains. Parasite. 2012;19:249. 22910667 10.1051/parasite/2012193249 PMC3671442

[27] Sleda MA, Pitafi ZF, Song W, Oldfield E, Moreno SN. Lipophilic bisphosphonates reduced cyst burden and ameliorated hyperactivity of mice chronically infected with *Toxoplasma gondii*. MBio. 2024;15:e01756-01724. 39387586 10.1128/mbio.01756-24 PMC11558998

[28] Parasuraman S, Raveendran R, Kesavan R. Blood sample collection in small laboratory animals. J Pharmacol Pharmacother. 2010;1:87–93. 21350616 10.4103/0976-500X.72350 PMC3043327

[29] Kasper DC, Sadeghi K, Prusa A-R, Reischer GH, Kratochwill K, Förster-Waldl E, et al. Quantitative real-time polymerase chain reaction for the accurate detection of *Toxoplasma gondii* in amniotic fluid. Diagn Microbiol Infect Dis. 2009;63:10–5. 18990529 10.1016/j.diagmicrobio.2008.09.009

[30] Li Z, Liu Q-S, Hu J-J, Deng C-Q, Li T, Zheng W-B, et al. Spatiotemporal diffusion, colonization, and antibody responses in susceptible C57BL/6J mice orally infected with *Toxoplasma gondii* cysts. Vet Sci. 2025;12:212. 40266920 10.3390/vetsci12030212 PMC11945890

[31] Sina S, Mohammad JM, Reza S, Anita M, Soudabeh E, Hadi M. Determination of parasitic burden in the brain tissue of infected mice in acute toxoplasmosis after treatment by fluconazole combined with sulfadiazine and pyrimethamine. Eur J Med Res. 2021;26:65. 34193287 10.1186/s40001-021-00537-3 PMC8243906

[32] El Shanawany EE, Abdel-Rahman EH, Nemr WA, Hassan SE, Hassan NMF, Desouky HM, et al. Comparative evaluation of live attenuated and killed tachyzoites as vaccine candidates for toxoplasmosis. AMB Express. 2025;15:102. 40637955 10.1186/s13568-025-01889-3 PMC12246356

[33] Arafa FM, Hezema NN, Aljuhani A, Aouad MR, Hagar M, Zakaria A, et al. Isatin-1,2,3-triazole derivatives: synthesis, molecular docking and evaluation against acute experimental toxoplasmosis. Acta Trop. 2024;260:107471. 39542154 10.1016/j.actatropica.2024.107471

[34] Tanaka T, Maeda H, Matsuo T, Boldbattar D, Umemiya-Shirafuji R, Kume A, et al. Parasiticidal activity of *Haemaphysalis longicornis* longicin P4 peptide against *Toxoplasma gondii*. Peptides. 2012;34:242–50. 21849158 10.1016/j.peptides.2011.07.027

[35] Hezema NN, Eltarahony MM, Abdel Salam SA. Therapeutic and antioxidant potential of bionanofactory *Ochrobactrum* sp.-mediated magnetite and zerovalent iron nanoparticles against acute experimental toxoplasmosis. PLoS Negl Trop Dis. 2023;17:e0011655. 37801440 10.1371/journal.pntd.0011655 PMC10558077

[36] Sullivan WJ Jr, Jeffers V. Mechanisms of *Toxoplasma gondii* persistence and latency. FEMS Microbiol Rev. 2012;36:717–33. 22091606 10.1111/j.1574-6976.2011.00305.x PMC3319474

[37] Gharamti AA, Rao A, Pecen PE, Henao-Martínez AF, Franco-Paredes C, Montoya JG. Acute *Toxoplasma* dissemination with encephalitis in the era of biological therapies. Open Forum Infect Dis. 2018;5:259. 10.1093/ofid/ofy259 PMC6237240 30460322

[38] Hou S, Liu Y, Tang Y, Wu M, Guan J, Li X, et al. Anti-*Toxoplasma gondii* effect of two spider venoms *in vitro* and *in vivo*. Toxicon. 2019;166:9–14. 31103717 10.1016/j.toxicon.2019.05.003

[39] Borges IP, Castanheira LE, Barbosa BF, De Souza DL, Da Silva RJ, Mineo JR, et al. Anti-parasitic effect on *Toxoplasma gondii* induced by BnSP-7, a Lys49-phospholipase A2 homologue from *Bothrops pauloensis* venom. Toxicon. 2016;119:84–91. 27212627 10.1016/j.toxicon.2016.05.010

[40] Izidoro LF, Alves LM, Rodrigues VM, Silva DA, Mineo JR. *Bothrops pirajai* snake venom L-amino acid oxidase: in vitro effects on infection of *Toxoplasma gondii* in human foreskin fibroblasts. Rev Bras Farmacogn. 2011;21:477–85.

[41] Bastos LM, Júnior RJ, Silva DA, Mineo JR, Vieira CU, Teixeira DN, et al. *Toxoplasma gondii*: effects of neuwiedase, a metalloproteinase from *Bothrops neuwiedi* snake venom, on the invasion and replication of human fibroblasts in vitro. Exp Parasitol. 2008;120:391–6. 18823981 10.1016/j.exppara.2008.09.008

[42] Allane D, Oussedik-Oumehdi H, Harrat Z, Seve M, Laraba-Djebari F. Isolation and characterization of an anti-leishmanial disintegrin from *Cerastes cerastes* venom. J Biochem Mol Toxicol. 2018;32:e22018. 10.1002/jbt.22018 29278277

[43] Liu Y, Tang Y, Tang X, Wu M, Hou S, Liu X, et al. Anti-*Toxoplasma gondii* effects of a novel spider peptide XYP1 *in vitro* and *in vivo*. Biomedicines. 2021;9:934. 34440138 10.3390/biomedicines9080934 PMC8392294

[44] Samy RP, Gopalakrishnakone P, Chow VT, Ho B. Viper metalloproteinase (*Agkistrodon halys pallas*) with antimicrobial activity against multi-drug resistant human pathogens. J Cell Physiol. 2008;216:54–68. 18297685 10.1002/jcp.21373

[45] Al-Kaysi M. Biochemical studies on the effect of *Toxoplasma* infection on liver and kidney functions in mice. Egypt J Comp Path Clin Path. 2010;23:174–85.

[46] Snyder LS, Heigh RI, Anderson ML. Cyclophosphamide-induced hepatotoxicity in a patient with Wegener’s granulomatosis. Mayo Clin Proc. 1993;68:1203–4. 8246624 10.1016/s0025-6196(12)60074-3

[47] Da Silva AS, Tonin AA, Thorstenberg ML, Leal DB, Fighera R, Flores MM, et al. Relationship between butyrylcholinesterase activity and liver injury in mice acute infected with *Toxoplasma gondii*. Pathol Res Pract. 2013;209:95–8. 23313104 10.1016/j.prp.2012.10.007

[48] Al-Jowari S, Hussein D. Effect of toxoplasmosis infection on liver and kidney functions among pregnant women in Abo-Gharib district-Iraq. Iraqi J Sci. 2014;55:101–5.

[49] El-Hamid A, Omnia M, El-Shaboury A, Baraka A, Khater H. Biochemical and histopahthological changes on *Toxoplasma* infected rats treated with lincocin and/ or green tea. Benha Vet Med J. 2018;35:216–27.

[50] Salama S, Al-Sadoon M. The possible role of low doses of *Cerastes cerastes* crude venom in mitigating doxorubicin induced oxidative damage in male rats. J Rad Res Appl Sci. 2012;5:408–20.

[51] Branten AJ, Reichert LJ, Koene RA, Wetzels JF. Oral cyclophosphamide versus chlorambucil in the treatment of patients with membranous nephropathy and renal insufficiency. QJM. 1998;91:359–66. 9709470 10.1093/qjmed/91.5.359

[52] Li J, Pan L, Pan W, Li N, Tang B. Recent progress of oxidative stress associated biomarker detection. Chem Commun. 2023;59:7361–74. 10.1039/d3cc00878a 37194341

[53] Kim JH, Lee J, Bae SJ, Kim Y, Park BJ, Choi JW, et al. NADPH oxidase 4 is required for the generation of macrophage migration inhibitory factor and host defense against *Toxoplasma gondii* infection. Sci Rep. 2017;7:6361. 28743960 10.1038/s41598-017-06610-4 PMC5526938

[54] Yeo SJ, Jin C, Kim S, Park H. *In vitro* and *in vivo* effects of nitrofurantoin on experimental toxoplasmosis. Korean J Parasitol. 2016;54:155–61. 27180573 10.3347/kjp.2016.54.2.155 PMC4870977

[55] Ziba HAN, Mohammad M, Maryam B, Faeze F-P. *Toxoplasma gondii*: a possible inducer of oxidative stress in reproductive system of male rats. Iran J Parasitol. 2020;15:521–9. 33884009 10.18502/ijpa.v15i4.4857 PMC8039477

[56] Haffor AS, Al-Sadoon MK. Increased antioxidant potential and decreased free radical production in response to mild injection of crude venom, *Cerastes cerastes gasperetti*. Toxicol Mech Methods. 2008;18:11–6. 20020886 10.1080/15376510701728455

