# Supplementary material for: Evaluation of the potential therapeutic efficacy of Cerastes cerastes venom in acute experimental toxoplasmosis
Source: Parasit Vectors. 2026 Jan 24;19:87. doi: 10.1186/s13071-025-07209-9 (PMC12914995; doi:10.1186/s13071-025-07209-9)
Supplement: Supplementary file 1 — Additional file 1. Pilot study [file 13071_2025_7209_MOESM1_ESM.docx]

**Schedule of preliminary study**

**Animal grouping and experimental design**

Sixty-six mice were equally divided into three main groups: Group I, non-infected non-treated six control mice (NI-NT CTL), Group II, infected non-treated control (INF-NT CTL) consisting of six mice; Group III comprising fifty-four infected treated (INF-T) mice, which was further subdivided into three subgroups, a, b, and c according to the dose of *C. cerastes* venom (CCV). Each of these subgroups was further divided into three additional subgroups: 1, 2, and 3, based on the duration of exposure to CCV as follows:

Subgroup IIIa1: INF-T with 1/2 LD50 of CCV for one day.

Subgroup IIIa2: INF-T with 1/2 LD50 of CCV for three days.

Subgroup IIIa3: INF-T with 1/2 LD50 of CCV for six days.

Subgroup IIIb1: INF-T with 1/4 LD50 of CCV for one day.

Subgroup IIIb2: INF-T with 1/4 LD50 of CCV for three days.

Subgroup IIIb3: INF-T with 1/4 LD50 of CCV for six days.

Subgroup IIIc1: INF-T with 1/10 LD50 of CCV for one day.

Subgroup IIIc2: INF-T with 1/10 LD50 of CCV for three days.

Subgroup IIIc3: INF-T with 1/10 LD50 of CCV for six days.

Each mouse in groups II and III was IP inoculated with 5×10^3^ tachyzoites of the RH HXGPRT (-) virulent strain of *T. gondii*. The LD50 of CCV was determined to be 0.535 mg/kg. Treatment started six hours after the infection, and animals were sacrificed on the seventh day post-infection.

**Assessment of the therapeutic efficacy of CCV against *T. gondii:***

1. **Parasitological study**

The peritoneal fluid containing tachyzoites was collected from each infected mouse on the day of sacrifice. The mean number of extracellular tachyzoites was calculated per ml of peritoneal fluid in each treated subgroup and compared with their corresponding INF-NT CTL (group II). Then, the percentage reduction (% R) in the mean peritoneal parasite burden was estimated.

1. **Biochemical study**

Blood was collected from each mouse in all infected groups (II and III) on the day of sacrifice alongside the NI-NT CTL (group I). The sera were separated and used to verify the safety of the used dose of CCV by measuring liver function markers (alanine transaminase [ALT] and aspartate transaminase [AST]), kidney function markers (urea and creatinine), oxidative stress markers (malondialdehyde [MDA] and reduced glutathione [GSH]) in comparison with their corresponding NI-NT CTL.

**The effect of different doses of** **CCV given at different durations on the peritoneal parasite burden in the infected treated mice in comparison to their corresponding infected non-treated control**

| **Group**    **Peritoneal  parasite burden**  **(1 × 10^4^)** | **II**  **(INF-NT CTL)** | **III**  **(INF-T)** | | | | | | | | |
| --- | --- | --- | --- | --- | --- | --- | --- | --- | --- | --- |
|  |  | **Subgroup IIIa**  **INF-T with 1/2 LD50** | | | **Subgroup IIIb**  **INF-T with 1/4 LD50** | | | **Subgroup IIIc**  **INF-T with 1/10 LD50** | | |
|  |  | **IIIa1**  **(1 day)** | **IIIa2**  **(3 days)** | **IIIa3**  **(6 days)** | **IIIb1**  **(1 day)** | **IIIb2**  **(3 days)** | **IIIb3**  **(6 days)** | **IIIc1**  **(1 day)** | **IIIc2**  **(3 days)** | **IIIc3**  **(6 days)** |
| **Mean ± SD** | 493.0± 51.71 | 204.2 ± 41.58 | 48.62 ± 5.87 | 36.90 ± 13.87 | 251.5 ± 44.10 | 49.98 ± 17.39 | 38.40 ± 15.07 | 356.8 ± 30.67 | 137.3 ± 15.08 | 88.50 ± 28.70 |
| **% R** |  | **58.58%** | **90.14%** | **92.51%** | **48.98%** | **89.86%** | **92.21%** | **27.63%** | **72.15%** | **82.04%** |
| ***p*-value compared to Group II** |  | <0.001^*^ | <0.001^*^ | <0.001^*^ | <0.001^*^ | <0.001^*^ | <0.001^*^ | 0.001^*^ | <0.001^*^ | <0.001^*^ |
| ***p*-value between IIIa1 & IIIa2** |  | <0.001^*^ | |  |  |  |  |  |  |  |
| ***p*-value between IIIa1 & IIIa3** |  | <0.001^*^ | | |  |  |  |  |  |  |
| ***p*-value between IIIa2 & IIIa3** |  |  | 1.000 | |  |  |  |  |  |  |
| ***p*-value between IIIb1 & IIIb2** |  |  |  |  | <0.001^*^ | |  |  |  |  |
| ***p*-value between IIIb1 & IIIb3** |  |  |  |  | <0.001^*^ | | |  |  |  |
| ***p*-value between IIIb2 & IIIb3** |  |  |  |  |  | 1.000 | |  |  |  |
| ***p*-value between IIIc1 & IIIc2** |  |  |  |  |  |  |  | <0.001^*^ | |  |
| ***p*-value between IIIc1 & IIIc3** |  |  |  |  |  |  |  | <0.001^*^ | | |
| ***p*-value between IIIc2 & IIIc3** |  |  |  |  |  |  |  |  | 0.619 | |
| ***p*-value between IIIa1 & IIIb1** |  | 0.656 | | | |  |  |  |  |  |
| ***p*-value between IIIa1 & IIIc1** |  | <0.001^*^ | | | | | | |  |  |
| ***p*-value between IIIb1 & IIIc1** |  |  |  |  | 0.011^*^ | | | |  |  |
| ***p*-value between IIIa2 & IIIb2** |  |  | 1.000 | | | |  |  |  |  |
| ***p*-value between IIIa2 & IIIc2** |  |  | 0.044^*^ | | | | | | |  |
| ***p*-value between IIIb2 & IIIc2** |  |  |  |  |  | 0.049^*^ | | | |  |
| ***p*-value between IIIa3 & IIIb3** |  |  |  | 1.000 | | | |  |  |  |
| ***p*-value between IIIa3 & IIIc3** |  |  |  | 0.550 | | | | | | |
| ***p*-value between IIIb3 & IIIc3** |  |  |  |  |  |  | 0.587 | | | |

**One-way ANOVA** was used, followed by **Post Hoc** test **(Tukey)** for pairwise comparison

*: Statistically significant at p ≤ 0.05

**The effect of different doses of CCV given at different durations on the serum ALT level in the infected treated mice in comparison to their controls**

| **Group**  **Serum ALT**  **(u/ml)** | **I**  **(NI-NT)** | **II**  **(INF-NT)** | **III**  **(INF-T)** | | | | | | | | |
| --- | --- | --- | --- | --- | --- | --- | --- | --- | --- | --- | --- |
|  |  |  | **Subgroup IIIa**  **INF-T with 1/2 LD50** | | | **Subgroup IIIb**  **INF-T with 1/4 LD50** | | | **Subgroup IIIc**  **INF-T with 1/10 LD50** | | |
|  |  |  | **IIIa1**  **(1 day)** | **IIIa2**  **(3 days)** | **IIIa3**  **(6 days)** | **IIIb1**  **(1 day)** | **IIIb2**  **(3 days)** | **IIIb3**  **(6 days)** | **IIIc1**  **(1 day)** | **IIIc2**  **(3 days)** | **IIIc3**  **(6 days)** |
| **Mean ± SD** | 24.8 ± 2.8 | 89.3±6.7 | 32.12 ± 4.3 | 41.72 ± 12.4 | 53.2 ± 2.6 | 28.34 ±3.1 | 29.42 ±7.3 | 41.82 ±4.3 | 26.87 ± 3.6 | 28.47 ± 2.1 | 39.80 ± 1.9 |
| ***p*-value compared to Group I** |  | <0.001* | 1.000 | 0.511 | 0.011 | 1.000 | 1.000 | 0.496 | 1.000 | 1.000 | 0.744 |
| ***p*-value compared to Group II** |  |  | <0.001* | <0.001* | 0.001* | <0.001* | <0.001* | <0.001* | <0.001* | <0.001* | <0.001* |
| ***p*-value between IIIa1 & IIIa2** |  |  | 0.625 | |  |  |  |  |  |  |  |
| ***p*-value between IIIa1 & IIIa3** |  |  | 0.167 | | |  |  |  |  |  |  |
| ***p*-value between IIIa2 & IIIa3** |  |  |  | 0.521 | |  |  |  |  |  |  |
| ***p*-value between IIIb1 & IIIb2** |  |  |  |  |  | 0.985 | |  |  |  |  |
| ***p*-value between IIIb1 & IIIb3** |  |  |  |  |  | 0.179 | | |  |  |  |
| ***p*-value between IIIb2 & IIIb3** |  |  |  |  |  |  | 0.220 | |  |  |  |
| ***p*-value between IIIc1 & IIIc2** |  |  |  |  |  |  |  |  | 0.878 | |  |
| ***p*-value between IIIc1 & IIIc3** |  |  |  |  |  |  |  |  | 0.014* | | |
| ***p*-value between IIIc2 & IIIc3** |  |  |  |  |  |  |  |  |  | 0.024* | |
| ***p*-value between IIIa1 & IIIb1** |  |  | 0.721 | | | |  |  |  |  |  |
| ***p*-value between IIIa1 & IIIc1** |  |  | 0.549 | | | | | | |  |  |
| ***p*-value between IIIb1 & IIIc1** |  |  |  |  |  | 0.950 | | | |  |  |
| ***p*-value between IIIa2 & IIIb2** |  |  |  | 0.524 | | | |  |  |  |  |
| ***p*-value between IIIa2 & IIIc2** |  |  |  | 0.478 | | | | | | |  |
| ***p*-value between IIIb2 & IIIc2** |  |  |  |  |  |  | 0.996 | | | |  |
| ***p*-value between IIIa3 & IIIb3** |  |  |  |  | 0.059 | | | |  |  |  |
| ***p*-value between IIIa3 & IIIc3** |  |  |  |  | 0.038* | | | | | | |
| ***p*-value between IIIb3 & IIIc3** |  |  |  |  |  |  |  | 0.936 | | | |

**One-way ANOVA** was used, followed by **Post Hoc** test **(Tukey)** for pairwise comparison

*: Statistically significant at p ≤ 0.05

**The effect of different doses of CCV given at different durations on the serum AST level in the infected treated mice in comparison to their controls**

| **Group**  **Serum AST**  **(u/ml)** | **I**  **(NI-NT)** | **II**  **(INF-NT)** | **III**  **(INF-T)** | | | | | | | | |
| --- | --- | --- | --- | --- | --- | --- | --- | --- | --- | --- | --- |
|  |  |  | **Subgroup IIIa**  **INF-T with 1/2 LD50** | | | **Subgroup IIIb**  **INF-T with 1/4 LD50** | | | **Subgroup IIIc**  **INF-T with 1/10 LD50** | | |
|  |  |  | **IIIa1**  **(1 day)** | **IIIa2**  **(3 days)** | **IIIa3**  **(6 days)** | **IIIb1**  **(1 day)** | **IIIb2**  **(3 days)** | **IIIb3**  **(6 days)** | **IIIc1**  **(1 day)** | **IIIc2**  **(3 days)** | **IIIc3**  **(6 days)** |
| **Mean ± SD** | 31.26 ± 6.3 | 84.63± 6.3 | 40.31 ± 0.9 | 58.68 ± 0.51 | 63.40 ± 1.2 | 36.13± 1.4 | 41.26 ± 3.4 | 56.85 ± 6.8 | 31.97 ± 0.5 | 32.27 ± 3.4 | 53.20 ± 2.6 |
| ***p*-value compared to Group I** |  | <0.001* | 1.000 | <0.001* | <0.001* | 1.000 | 0.740 | <0.001* | 1.000 | 1.000 | 0.001* |
| ***p*-value compared to Group II** |  |  | <0.001* | 0.006* | 0.076 | <0.001* | <0.001* | 0.002* | <0.001* | <0.001* | <0.001* |
| ***p*-value between IIIa1 & IIIa2** |  |  | <0.001* | |  |  |  |  |  |  |  |
| ***p*-value between IIIa1 & IIIa3** |  |  | <0.001* | | |  |  |  |  |  |  |
| ***p*-value between IIIa2 & IIIa3** |  |  |  | 0.017* | |  |  |  |  |  |  |
| ***p*-value between IIIb1 & IIIb2** |  |  |  |  |  | 0.665 | |  |  |  |  |
| ***p*-value between IIIb1 & IIIb3** |  |  |  |  |  | 0.027* | | |  |  |  |
| ***p*-value between IIIb2 & IIIb3** |  |  |  |  |  |  | 0.079 | |  |  |  |
| ***p*-value between IIIc1 & IIIc2** |  |  |  |  |  |  |  |  | 0.995 | |  |
| ***p*-value between IIIc1 & IIIc3** |  |  |  |  |  |  |  |  | 0.001* | | |
| ***p*-value between IIIc2 & IIIc3** |  |  |  |  |  |  |  |  |  | 0.002* | |
| ***p*-value between IIIa1 & IIIb1** |  |  | 0.041* | | | |  |  |  |  |  |
| ***p*-value between IIIa1 & IIIc1** |  |  | 0.002* | | | | | | |  |  |
| ***p*-value between IIIb1 & IIIc1** |  |  |  |  |  | 0.042* | | | |  |  |
| ***p*-value between IIIa2 & IIIb2** |  |  |  | 0.007* | | | |  |  |  |  |
| ***p*-value between IIIa2 & IIIc2** |  |  |  | 0.001* | | | | | | |  |
| ***p*-value between IIIb2 & IIIc2** |  |  |  |  |  |  | 0.103 | | | |  |
| ***p*-value between IIIa3 & IIIb3** |  |  |  |  | 0.500 | | | |  |  |  |
| ***p*-value between IIIa3 & IIIc3** |  |  |  |  | 0.231 | | | | | | |
| ***p*-value between IIIb3 & IIIc3** |  |  |  |  |  |  |  | 0.792 | | | |

**One-way ANOVA** was used, followed by **Post Hoc** test **(Tukey)** for pairwise comparison

*: Statistically significant at p ≤ 0.05

**The effect of different doses of CCV given at different durations on the serum urea level in the infected treated mice in comparison to their controls**

| **Group**  **Serum Urea**  **(mg/dl)** | **I**  **(NI-NT)** | **II**  **(INF-NT)** | **III**  **(INF-T)** | | | | | | | | |
| --- | --- | --- | --- | --- | --- | --- | --- | --- | --- | --- | --- |
|  |  |  | **Subgroup IIIa**  **INF-T with 1/2 LD50** | | | **Subgroup IIIb**  **INF-T with 1/4 LD50** | | | **Subgroup IIIc**  **INF-T with 1/10 LD50** | | |
|  |  |  | **IIIa1**  **(1 day)** | **IIIa2**  **(3 days)** | **IIIa3**  **(6 days)** | **IIIb1**  **(1 day)** | **IIIb2**  **(3 days)** | **IIIb3**  **(6 days)** | **IIIc1**  **(1 day)** | **IIIc2**  **(3 days)** | **IIIc3**  **(6 days)** |
| **Mean ± SD** | 16.33± 3.8 | 63.67 ±8.9 | 34.21± 4.5 | 49.34 ± 1.2 | 53.12 ±0.2 | 29.11 ±1.2 | 33.04 ± 0.2 | 41.25±1.7 | 19.71±0.5 | 23.26±0.5 | 27.91±2.6 |
| ***p*-value compared to Group I** |  | <0.001* | 0.010* | <0.001* | <0.001* | 0.161 | 0.019* | <0.001* | 1.000 | 1.000 | 0.299 |
| ***p*-value compared to Group II** |  |  | <0.001* | 0.070 | 0.500 | <0.001* | <0.001* | 0.001* | <0.001* | <0.001* | <0.001* |
| ***p*-value between IIIa1 & IIIa2** |  |  | 0.011* | |  |  |  |  |  |  |  |
| ***p*-value between IIIa1 & IIIa3** |  |  | 0.004* | | |  |  |  |  |  |  |
| ***p*-value between IIIa2 & IIIa3** |  |  |  | 0.555 | |  |  |  |  |  |  |
| ***p*-value between IIIb1 & IIIb2** |  |  |  |  |  | 0.096 | |  |  |  |  |
| ***p*-value between IIIb1 & IIIb3** |  |  |  |  |  | 0.001* | | |  |  |  |
| ***p*-value between IIIb2 & IIIb3** |  |  |  |  |  |  | 0.004* | |  |  |  |
| ***p*-value between IIIc1 & IIIc2** |  |  |  |  |  |  |  |  | 0.258 | |  |
| ***p*-value between IIIc1 & IIIc3** |  |  |  |  |  |  |  |  | 0.015* | | |
| ***p*-value between IIIc2 & IIIc3** |  |  |  |  |  |  |  |  |  | 0.129 | |
| ***p*-value between IIIa1 & IIIb1** |  |  | 0.372 | | | |  |  |  |  |  |
| ***p*-value between IIIa1 & IIIc1** |  |  | 0.014* | | | | | | |  |  |
| ***p*-value between IIIb1 & IIIc1** |  |  |  |  |  | 0.080 | | | |  |  |
| ***p*-value between IIIa2 & IIIb2** |  |  |  | <0.001* | | | |  |  |  |  |
| ***p*-value between IIIa2 & IIIc2** |  |  |  | <0.001* | | | | | | |  |
| ***p*-value between IIIb2 & IIIc2** |  |  |  |  |  |  | <0.001* | | | |  |
| ***p*-value between IIIa3 & IIIb3** |  |  |  |  | 0.005* | | | |  |  |  |
| ***p*-value between IIIa3 & IIIc3** |  |  |  |  | <0.001* | | | | | | |
| ***p*-value between IIIb3 & IIIc3** |  |  |  |  |  |  |  | 0.003* | | | |

**One-way ANOVA** was used, followed by **Post Hoc** test **(Tukey)** for pairwise comparison

*: Statistically significant at p ≤ 0.05

**The effect of different doses of CCV given at different durations on the serum creatinine level in the infected treated mice in comparison to their controls**

| **Group**  **Serum Creatinine**  **(mg/dl)** | **I**  **(NI-NT)** | **II**  **(INF-NT)** | **III**  **(INF-T)** | | | | | | | | |
| --- | --- | --- | --- | --- | --- | --- | --- | --- | --- | --- | --- |
|  |  |  | **Subgroup IIIa**  **INF-T with 1/2 LD50** | | | **Subgroup IIIb**  **INF-T with 1/4 LD50** | | | **Subgroup IIIc**  **INF-T with 1/10 LD50** | | |
|  |  |  | **IIIa1**  **(1 day)** | **IIIa2**  **(3 days)** | **IIIa3**  **(6 days)** | **IIIb1**  **(1 day)** | **IIIb2**  **(3 days)** | **IIIb3**  **(6 days)** | **IIIc1**  **(1 day)** | **IIIc2**  **(3 days)** | **IIIc3**  **(6 days)** |
| **Mean ± SD** | 0.67±0.2 | 0.91 ±0.3 | 0.82±0.4 | 0.91±0.1 | 0.96±0.2 | 0.74±0.38 | 0.89±0.1 | 0.92±0.3 | 0.78±0.3 | 0.81±0.2 | 0.87±0.1 |
| ***p*-value compared to Group I** |  | 1.000 | 1.000 | 1.000 | 1.000 | 1.000 | 1.000 | 1.000 | 1.000 | 1.000 | 1.000 |
| ***p*-value compared to Group II** |  |  | 1.000 | 1.000 | 1.000 | 1.000 | 1.000 | 1.000 | 1.000 | 1.000 | 1.000 |
| ***p*-value between IIIa1 & IIIa2** |  |  | 0.559 | |  |  |  |  |  |  |  |
| ***p*-value between IIIa1 & IIIa3** |  |  | 0.684 | | |  |  |  |  |  |  |
| ***p*-value between IIIa2 & IIIa3** |  |  |  | 0.973 | |  |  |  |  |  |  |
| ***p*-value between IIIb1 & IIIb2** |  |  |  |  |  | 0.913 | |  |  |  |  |
| ***p*-value between IIIb1 & IIIb3** |  |  |  |  |  | 0.878 | | |  |  |  |
| ***p*-value between IIIb2 & IIIb3** |  |  |  |  |  |  | 0.996 | |  |  |  |
| ***p*-value between IIIc1 & IIIc2** |  |  |  |  |  |  |  |  | 0.994 | |  |
| ***p*-value between IIIc1 & IIIc3** |  |  |  |  |  |  |  |  | 0.945 | | |
| ***p*-value between IIIc2 & IIIc3** |  |  |  |  |  |  |  |  |  | 0.975 | |
| ***p*-value between IIIa1 & IIIb1** |  |  | 0.569 | | | |  |  |  |  |  |
| ***p*-value between IIIa1 & IIIc1** |  |  | 0.626 | | | | | | |  |  |
| ***p*-value between IIIb1 & IIIc1** |  |  |  |  |  | 0.994 | | | |  |  |
| ***p*-value between IIIa2 & IIIb2** |  |  |  | 0.994 | | | |  |  |  |  |
| ***p*-value between IIIa2 & IIIc2** |  |  |  | 0.855 | | | | | | |  |
| ***p*-value between IIIb2 & IIIc2** |  |  |  |  |  |  | 0.903 | | | |  |
| ***p*-value between IIIa3 & IIIb3** |  |  |  |  | 0.989 | | | |  |  |  |
| ***p*-value between IIIa3 & IIIc3** |  |  |  |  | 0.945 | | | | | | |
| ***p*-value between IIIb3 & IIIc3** |  |  |  |  |  |  |  | 0.982 | | | |

**One-way ANOVA** was used, followed by **Post Hoc** test **(Tukey)** for pairwise comparison

*: Statistically significant at p ≤ 0.05

**The effect of different doses of CCV given at different durations on the serum MDA level in the infected treated mice in comparison to their controls**

| **Group**  **Serum MDA**  **(****nmol/ml)** | **I**  **(NI-NT)** | **II**  **(INF-NT)** | **III**  **(INF-T)** | | | | | | | | |
| --- | --- | --- | --- | --- | --- | --- | --- | --- | --- | --- | --- |
|  |  |  | **Subgroup IIIa**  **INF-T with 1/2 LD50** | | | **Subgroup IIIb**  **INF-T with 1/4 LD50** | | | **Subgroup IIIc**  **INF-T with 1/10 LD50** | | |
|  |  |  | **IIIa1**  **(1 day)** | **IIIa2**  **(3 days)** | **IIIa3**  **(6 days)** | **IIIb1**  **(1 day)** | **IIIb2**  **(3 days)** | **IIIb3**  **(6 days)** | **IIIc1**  **(1 day)** | **IIIc2**  **(3 days)** | **IIIc3**  **(6 days)** |
| **Mean ± SD** | 9.10 ± 0.90 | 14.98 ± 1.36 | 11.32 ± 0.50 | 13.54 ± 0.70 | 13.88 ± 0.90 | 11.31 ± 0.22 | 10.42 ± 0.46 | 11.15 ± 0.53 | 11.31 ± 0.63 | 11.38 ± 0.53 | 12.79 ± 0.56 |
| ***p*-value compared to Group I** |  | <0.001^*^ | 0.034^*^ | <0.001^*^ | <0.001^*^ | 0.035^*^ | 0.504 | 0.061 | 0.035^*^ | 0.027^*^ | <0.001^*^ |
| ***p*-value compared to Group II** |  |  | <0.001^*^ | 0.386 | 0.732 | <0.001^*^ | <0.001^*^ | <0.001^*^ | <0.001^*^ | <0.001^*^ | 0.037^*^ |
| ***p*-value between IIIa1 & IIIa2** |  |  | 0.034^*^ | |  |  |  |  |  |  |  |
| ***p*-value between IIIa1 & IIIa3** |  |  | 0.009^*^ | | |  |  |  |  |  |  |
| ***p*-value between IIIa2 & IIIa3** |  |  |  | 1.000 | |  |  |  |  |  |  |
| ***p*-value between IIIb1 & IIIb2** |  |  |  |  |  | 0.902 | |  |  |  |  |
| ***p*-value between IIIb1 & IIIb3** |  |  |  |  |  | 1.000 | | |  |  |  |
| ***p*-value between IIIb2 & IIIb3** |  |  |  |  |  |  | 0.970 | |  |  |  |
| ***p*-value between IIIc1 & IIIc2** |  |  |  |  |  |  |  |  | 1.000 | |  |
| ***p*-value between IIIc1 & IIIc3** |  |  |  |  |  |  |  |  | 0.351 | | |
| ***p*-value between IIIc2 & IIIc3** |  |  |  |  |  |  |  |  |  | 0.415 | |
| ***p*-value between IIIa1 & IIIb1** |  |  | 1.000 | | | |  |  |  |  |  |
| ***p*-value between IIIa1 & IIIc1** |  |  | 1.000 | | | | | | |  |  |
| ***p*-value between IIIb1 & IIIc1** |  |  |  |  |  | 1.000 | | | |  |  |
| ***p*-value between IIIa2 & IIIb2** |  |  |  | 0.001^*^ | | | |  |  |  |  |
| ***p*-value between IIIa2 & IIIc2** |  |  |  | 0.042^*^ | | | | | | |  |
| ***p*-value between IIIb2 & IIIc2** |  |  |  |  |  |  | 0.854 | | | |  |
| ***p*-value between IIIa3 & IIIb3** |  |  |  |  | 0.005^*^ | | | |  |  |  |
| ***p*-value between IIIa3 & IIIc3** |  |  |  |  | 0.742 | | | | | | |
| ***p*-value between IIIb3 & IIIc3** |  |  |  |  |  |  |  | 0.229 | | | |

**One-way ANOVA** was used, followed by **Post Hoc** test **(Tukey)** for pairwise comparison

*: Statistically significant at p ≤ 0.05

**The effect of different doses of CCV given at different durations on the serum GSH level in the infected treated mice in comparison to their controls**

| **Group**  **Serum GSH**  **(mg/dl)** | **I**  **(NI-NT)** | **II**  **(INF-NT)** | **III**  **(INF-T)** | | | | | | | | |
| --- | --- | --- | --- | --- | --- | --- | --- | --- | --- | --- | --- |
|  |  |  | **Subgroup IIIa**  **INF-T with 1/2 LD50** | | | **Subgroup IIIb**  **INF-T with 1/4 LD50** | | | **Subgroup IIIc**  **INF-T with 1/10 LD50** | | |
|  |  |  | **IIIa1**  **(1 day)** | **IIIa2**  **(3 days)** | **IIIa3**  **(6 days)** | **IIIb1**  **(1 day)** | **IIIb2**  **(3 days)** | **IIIb3**  **(6 days)** | **IIIc1**  **(1 day)** | **IIIc2**  **(3 days)** | **IIIc3**  **(6 days)** |
| **Mean ± SD** | 0.92 ± 0.04 | 0.90 ± 0.06 | 2.18 ± 0.15 | 2.79 ± 0.16 | 3.66 ± 0.28 | 1.70 ± 0.09 | 3.44 ± 0.30 | 4.56 ± 0.33 | 1.48 ± 0.10 | 1.67 ± 0.12 | 2.02 ± 0.11 |
| ***p*-value compared to Group I** |  | 1.000 | <0.001^*^ | <0.001^*^ | <0.001^*^ | 0.001^*^ | <0.001^*^ | <0.001^*^ | 0.039^*^ | 0.002^*^ | <0.001^*^ |
| ***p*-value compared to Group II** |  |  | <0.001^*^ | <0.001^*^ | <0.001^*^ | 0.001^*^ | <0.001^*^ | <0.001^*^ | 0.028^*^ | 0.002^*^ | <0.001^*^ |
| ***p*-value between IIIa1 & IIIa2** |  |  | 0.018^*^ | |  |  |  |  |  |  |  |
| ***p*-value between IIIa1 & IIIa3** |  |  | <0.001^*^ | | |  |  |  |  |  |  |
| ***p*-value between IIIa2 & IIIa3** |  |  |  | <0.001^*^ | |  |  |  |  |  |  |
| ***p*-value between IIIb1 & IIIb2** |  |  |  |  |  | <0.001^*^ | |  |  |  |  |
| ***p*-value between IIIb1 & IIIb3** |  |  |  |  |  | <0.001^*^ | | |  |  |  |
| ***p*-value between IIIb2 & IIIb3** |  |  |  |  |  |  | <0.001^*^ | |  |  |  |
| ***p*-value between IIIc1 & IIIc2** |  |  |  |  |  |  |  |  | 0.967 | |  |
| ***p*-value between IIIc1 & IIIc3** |  |  |  |  |  |  |  |  | 0.049^*^ | | |
| ***p*-value between IIIc2 & IIIc3** |  |  |  |  |  |  |  |  |  | 0.454 | |
| ***p*-value between IIIa1 & IIIb1** |  |  | 0.110 | | | |  |  |  |  |  |
| ***p*-value between IIIa1 & IIIc1** |  |  | 0.005^*^ | | | | | | |  |  |
| ***p*-value between IIIb1 & IIIc1** |  |  |  |  |  | 0.918 | | | |  |  |
| ***p*-value between IIIa2 & IIIb2** |  |  |  | 0.010^*^ | | | |  |  |  |  |
| ***p*-value between IIIa2 & IIIc2** |  |  |  | <0.001^*^ | | | | | | |  |
| ***p*-value between IIIb2 & IIIc2** |  |  |  |  |  |  | <0.001^*^ | | | |  |
| ***p*-value between IIIa3 & IIIb3** |  |  |  |  | <0.001^*^ | | | |  |  |  |
| ***p*-value between IIIa3 & IIIc3** |  |  |  |  | <0.001^*^ | | | | | | |
| ***p*-value between IIIb3 & IIIc3** |  |  |  |  |  |  |  | <0.001^*^ | | | |

**One-way ANOVA** was used, followed by **Post Hoc** test **(Tukey)** for pairwise comparison

*: Statistically significant at p ≤ 0.05
